# Sero-catalytic and spatial modeling of dengue virus force of infection in Timor-Leste

**DOI:** 10.1038/s41467-026-76716-9

**Published:** 2026-09-15

**Authors:** Paul Arkell, Filipe de Neri Machado, Anna Vicco, Victoria M. Cox, Maria Y. Tanesi, Deolindo Ximenes, Wes Hinsley, Frederico Bosco, Nelia Gomes, Tessa Oakley, Vanessa Solano, Sarah L. Sheridan, Michael David, Salvador Amaral, Luzia Freitas, Anthony DK Draper, Nevio Sarmento, Endang Soares da Silva, Lucsendar Alves, Neil Ferguson, Christl A. Donnelly, Jennifer Yan, Joshua R. Francis, Ilaria Dorigatti, Nelson Martins

**Affiliations:** 1 Global and Tropical Health Division, Menzies School of Health Research, Charles Darwin University, Dili, Timor-Leste; 2 Department of Infectious Diseases, Faculty of Medicine, Imperial College, London, UK; 3 Instituto Nacional de Saude Publica de Timor-Leste, Timor-Leste Ministry of Health, Dili, Timor-Leste; 4 Departmanto Vigilancia no Epidemiojia, Timor-Leste Ministry of Health, Dili, Timor-Leste; 5 MRC Centre for Global Infectious Disease Analysis, Department of Infectious Disease Epidemiology, School of Public Health, Imperial College, London, UK; 6 Research Institute for the Environment and Livelihoods, Charles Darwin University, Darwin, NT Australia; 7 National Centre for Immunisation Research and Surveillance (NCIRS), Sydney, NSW Australia; 8 Daffodil Centre, The University of Sydney, a joint venture with Cancer Council New South Wales, Sydney, NSW Australia; 9 School of Medicine & Dentistry, Griffith University, Gold Coast, QLD Australia; 10 Infectious Diseases Data Observatory, Oxford University, Oxford, UK; 11 Northern Territory Centre for Disease Control, Public Health Division, NT Health, Darwin, NT Australia; 12 National Centre for Epidemiology and Population Health, Australian National University, Canberra, ACT Australia; 13 Laboratório Nacional da Saúde, Dili, Timor-Leste; 14 Department of Statistics, University of Oxford, Oxford, UK; 15 Pandemic Sciences Institute, University of Oxford, Oxford, UK

**Keywords:** ELISA, Dengue virus, Policy and public health in microbiology, Disease prevention, Epidemiology

## Abstract

Dengue virus (DENV) infection causes significant morbidity and mortality across tropical and sub-tropical regions, including in Timor-Leste. In this study we present the results of a national, population-representative serological survey, used to determine the drivers of observed variations in DENV force-of-infection (FOI, i.e. the average annual per-capita risk of infection for a susceptible person) at high spatial resolution. Households were randomly selected from census data and occupants aged above one year were tested using the Panbio Indirect dengue IgG enzyme-linked immunosorbent assay between October 2021 and February 2023. Sero-catalytic models accounting for household effect were applied to estimate the DENV FOI in enumeration areas (EAs, roughly equivalent to neighbourhoods/villages). Spatial models were applied to the local FOI estimates to assess their associations with climate and environmental factors, and to predict the FOI across the rest of Timor-Leste. A total of 4780 individuals from 1755 households across 102 EAs participated. DENV seroprevalence and FOI estimates were highly spatially heterogeneous across Timor-Leste, ranging from 0.010 (95% Credible Interval (CrI): 0.005, 0.018) in EA 10104032 in Fatubossa, municipality Aileu, to 0.519 (0.295, 0.816) in EA 40403012 in Goulolo, municipality Bobonaro. Relative humidity was negatively associated with DENV FOI. The spatial models predicted highest DENV FOI along the Northern and Eastern coasts, with hotspots of transmission in Dili and Baucau cities. Findings will strengthen surveillance and guide the implementation of additional DENV control strategies such as *Wolbachia*-carrying mosquitoes (currently being deployed in some parts of Timor-Leste), or vaccination.

## Abstract (web summary)

Dengue is a public health concern in tropical and subtropical regions. Here, the authors present findings from a national, population-representative serological survey in Timor Leste and identify substantial spatial variation in force of infection.

## Introduction

Dengue is a mosquito-borne disease affecting over 100 million people across tropical and sub-tropical regions of the world annually^1^. Dengue virus (DENV) is transmitted to humans through the bite of the *Aedes* mosquito (*Ae. aegypti* or *Ae. albopictus*), both of which are present in Timor-Leste and also transmit Zika virus and chikungunya virus^2^. The four DENV serotypes provide temporary cross-protection and long-term homologous immunity against the infecting serotype; hence people living in hyperendemic countries, where the four serotypes circulate, can experience up to four DENV infections during their lifetime. Whilst most infections are asymptomatic or cause a mild, self-limiting illness which does not prompt healthcare-seeking, a minority of infections cause severe disease, for example dengue haemorrhagic fever (DHF) or dengue shock syndrome (DSS), which can be fatal^3^. There is epidemiological and immunological evidence that severe disease is more common during secondary infections due to antibody-dependent enhancement^3^.

Case-based DENV surveillance typically relies on the clinical identification and/or laboratory confirmation of suspected cases, and their notification to regional or national surveillance authorities. Case notification data do not capture all infections and are therefore affected by under-reporting, which can vary across locations due to spatial differences in healthcare seeking and access, resources and healthcare capacity. In comparison, seroprevalence surveys quantify the number of individuals with IgG antibodies against DENV, which identifies previous infections without being affected by under-reporting (or age-dependent biases in reporting), as antibody development and detection does not depend on the onset of symptoms nor the sensitivity of surveillance.

Sero-catalytic models applied to age-stratified IgG seroprevalence data can be used to estimate DENV force of infection (FOI), i.e. the annual per-capita risk of infection for a susceptible person, which is a fundamental epidemiological metric that provides a unifying framework to assess variations in the intensity of DENV transmission across locations. Assuming endemic transmission, age can be taken as a proxy of the time spent at risk of infection and hence age-dependent differences in seroprevalence can be used to estimate the FOI: in high-FOI settings infections occur early in life and hence there is a steep increase in seroprevalence during the early years, whereas in low-transmission settings, individuals generally live longer uninfected and there is a slower increase in seroprevalence with age that typically reaches lower levels overall^4^.

Sero-catalytic models have been used to estimate the DENV FOI from cross-sectional serological data collected in Thailand^4^, China^5^, Vietnam^6^, Indonesia^7^, Brazil^8^, Colombia^9^, Bangladesh^10^, the Philippines^11^, Singapore^12^, Mexico^13^, and several other endemic countries^14–16^. Median estimates vary significantly between geographical locations, from under 0.01 in several regions of Madagascar^17^, Laos^18^ and Taiwan^19^, up to 0.649 (95% CrI 0.245, 0.984) in Zihuatanejo, Mexico^13,15^. A small number of studies have estimated FOI for individual DENV serotypes using assays which differentiate antibody responses, although cross-reactivity poses challenges to the interpretation of serotype-specific antibody data^18,20^. A recent systematic review of age-stratified seroprevalence studies found only five papers with geographically disaggregated data available for five or more different locations, only one of which was conducted in Southeast Asia^15^. That study included 3194 children from 30 randomly selected urban areas in Indonesia^7^. There is a lack of studies estimating and investigating the drivers behind spatial variations in DENV FOI, especially at high spatial resolution, i.e. in large numbers of locations, and in rural populations.

Timor-Leste is a mountainous country in Southeast Asia with a highly dispersed, mainly rural population of 1.3 million people who experience significant barriers to accessing healthcare. Routine surveillance data indicate that DENV transmission is spatially clustered and seasonal, with cases typically peaking in the wet season, which lasts from December to April^2,21^. In January 2022, Timor-Leste experienced a major DENV outbreak with 1286 reported cases and 20 deaths, which led to high numbers of hospitalisations, especially in Dili and Manatuto municipalities^21,22^. However, due to spatial heterogeneities in healthcare access and case reporting, the true burden of DENV and the extent of transmission across the island, including in its many remote areas, is not yet known^2^. Aside from a small, unrepresentative pilot study conducted between December 2018 and January 2019, no serological surveillance of DENV has occurred in Timor-Leste prior to the study presented in this paper^23^.

Here, the results of a national, population-representative serosurvey aimed to measure DENV seroprevalence and estimate the FOI at high spatial resolution across Timor-Leste are presented. The results were used to assess how climatic and environmental variables may contribute to the observed variation in DENV transmission intensity and reconstruct the expected seroprevalence across the rest of the country. Given the imminent deployment of *Wolbachia*-carrying mosquitoes^24^, the latest WHO recommendations on DENV vaccine use and the ongoing assessment of disease control interventions— including the potential use of the Qdenga (TAK-003) vaccine—this study provides valuable evidence to inform public health policy decisions^25,26^.

## Results

### Participants and samples

**Fig. 1:**
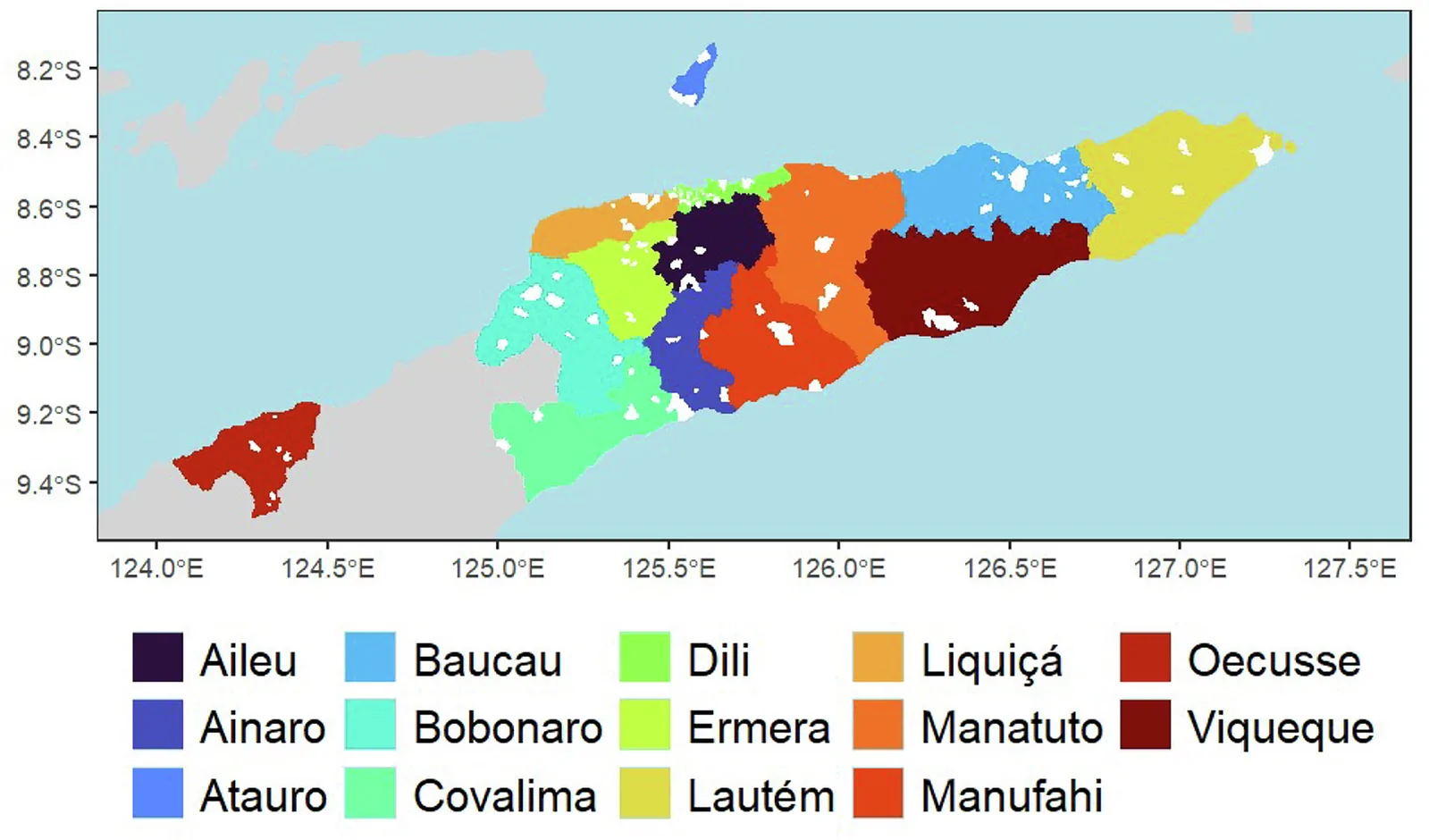
Map of the study area. The map shows the 102 enumeration areas (EAs) which were sampled during the serosurvey (in white), the municipalities of Timor-Leste the EAs belong to (in colour), Indonesian land (grey) and the Indian Ocean (pale blue).

**Fig. 2:**
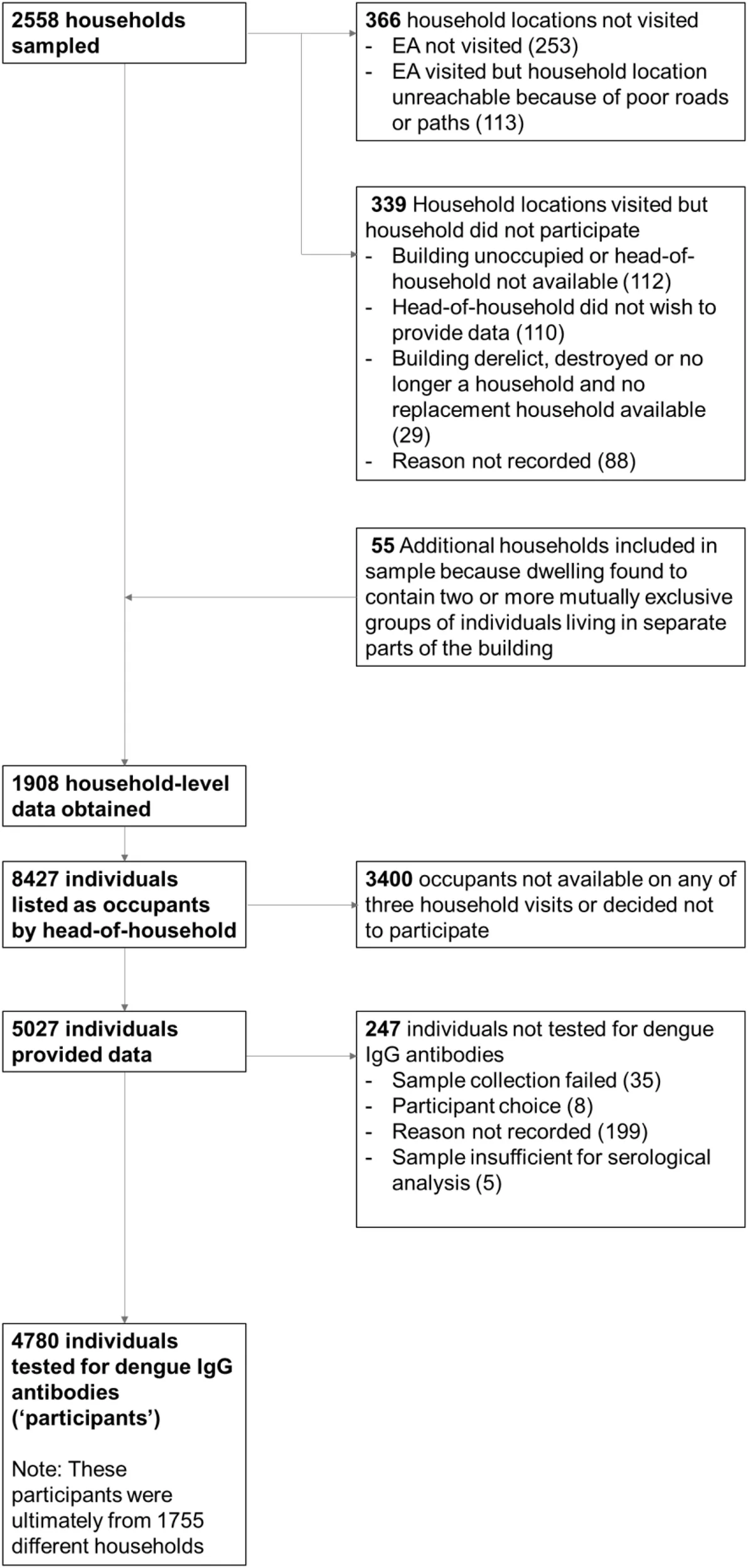
Flow diagram showing household- and individual-level participation in the study, and reasons for non-participation.

Between October 2021 and February 2023, 102 out of 113 selected EAs were visited (EA participation rate = 90.3%), with all 13 municipalities of Timor-Leste being represented (Fig. 1). At the time of the serosurvey, Atauro Island was part of Dili municipality. Eight EAs were not visited because of poor weather and road damage, two because of inadequate time, and one due to field staff illness. Occupants from 1755 out of 2558 selected households participated (household participation rate = 67.2%). Four thousand seven-hundred-and-eighty occupants provided a serum sample, which was analysed for dengue IgG antibodies (‘participants’). These were among 8427 individuals listed as household occupants (individual participation rate = 56.7%). A flow diagram showing household- and individual-level participation in the study, and reasons for non-participation, is shown in Fig. 2. EA-, household- and individual- participation rates in each municipality are shown in Table S3.

**Fig. 3:**
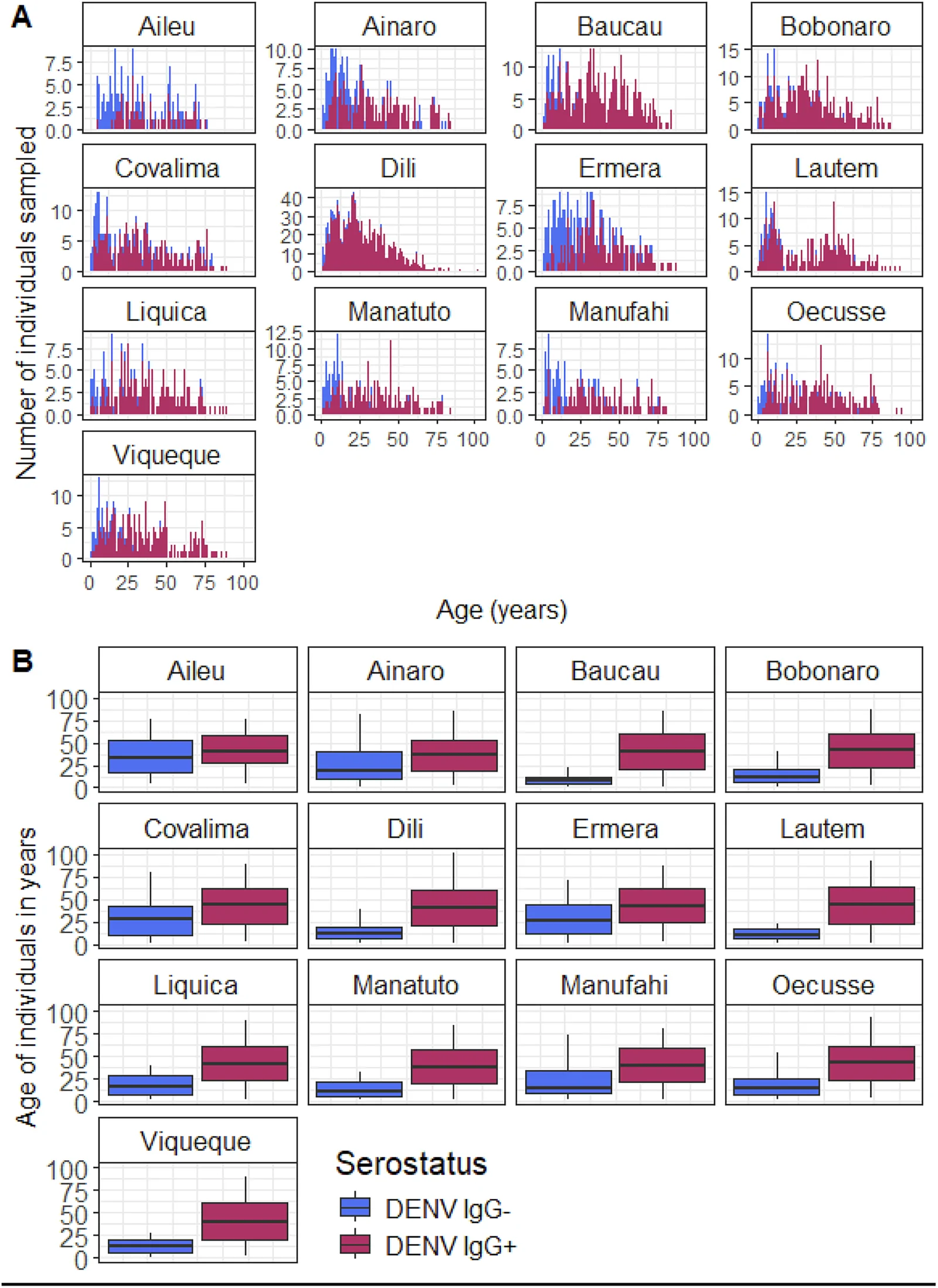
Serosurvey results by age. **A** The number and age of individuals sampled in each municipality, coloured by their dengue virus (DENV) serostatus as assessed by the Panbio Indirect dengue IgG enzyme-linked immunosorbent assay (ELISA). **B** Median (horizontal line), inter-quartile range (IQR, box), and the lowest and highest values within 1.5 × IQR from the box (error bar) of the age of the sampled individuals in each municipality, coloured by serostatus. For each municipality, the sample size of positive IgG samples out of total samples was: Aileu 44/93, Ainaro 62/99, Baucau 80/96, Bobonaro 78/98, Covalima 78/115, Dili 80/105, Ermera 70/119, Lautém 76/93, Liquiçá 72/95, Manatuto 75/95, Manufahi 60/87, Oecusse 72/97 and Viqueque 76/98.

The median age of participants was 28 years (inter-quartile range, IQR 13–46 years), and 59.2% were female. Most participants (73.0%) lived in a ‘rural’ household (as assigned during the 2015 national census). Three thousand, nine hundred thirty-one (83.1%) participants’ samples were IgG positive. Of these, 57 (1.2% of the whole dataset) had IV 0.9-1.1. Table S4 shows the demographic details of participants, and Fig. 3 illustrates the age distribution of the DENV IgG seronegative and seropositive individuals by municipality.

### Sero-catalytic modelling of DENV FOI

Figure S10 illustrates the fit of the sero-catalytic models to the observed clustered, age-stratified seroprevalence data from each of the 102 sampled EAs in Timor-Leste.

**Fig. 4:**
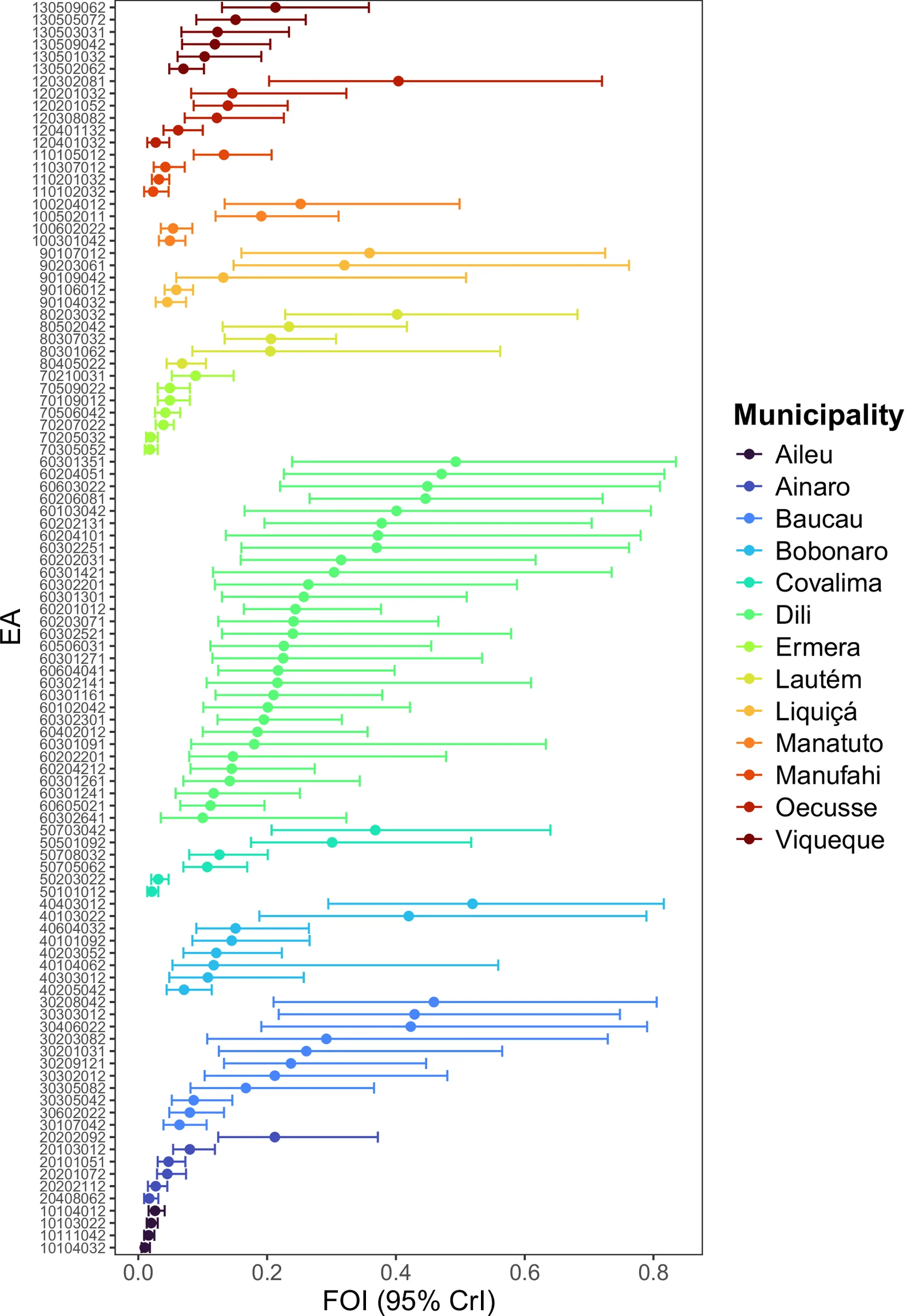
Estimated dengue virus (DENV) force of infection (FOI, the annual per-capita risk of infection for a susceptible person) in each enumeration area (EA). Estimated median (point) and 95% credible interval (CrI, error bar) of the total FOI, calculated from the whole posterior distribution obtained of 10,000 iterations, are coloured by the municipality of the EA.

Transmission intensity was heterogenous across the 102 EAs, with the FOI estimates ranging from a median of 0.010 (95% CrI: 0.005, 0.018) in EA 10104032 in Fatubossa, municipality Aileu, to a median of 0.519 (95% CrI: 0.295, 0.816) in EA 40403012 in Goulolo, municipality Bobonaro (see Table S5). The EAs in Bobonaro, Dili (including Atauro island) and Baucau municipalities had the highest FOI estimates, while those in Aileu, Ainaro and Ermera had the lowest estimates (Fig. 4).

The estimated test sensitivity and specificity were 99% (95% CrI: 99, 100%) and 98% (95% CrI: 95, 100%), respectively.

### Assessing drivers of DENV transmission

When spatial models were applied to the sampled FOI estimates, 88 of the 100 models contained one variable, 11 models contained two variables, and one model contained three variables. The variables included in the best-fitting models were relative humidity when temperature is at its minimum (chosen in 21 out of 88 single-variable models and two out of 11 two-variable models), relative humidity when temperature is at its maximum (17 out of 88 and two out of 11), and EVI (38 out of 88 and 10 out of the 11). These three variables were not collinear with each other (Fig. S11). The remaining variables were each chosen in less than 11 of all of the models. The joint posterior estimates for the beta coefficients showed that relative humidity was negatively associated with DENV FOI (median regression coefficient for relative humidity at minimum temperature = − 0.96, 95% CrI: −1.47, −0.39 and for relative humidity at maximum temperature = − 0.65, 95% CrI: −1.20, −0.13). Across the combined posterior distributions, EVI was not significantly associated with DENV FOI (median regression coefficient = 0.17, 95% CrI: −0.33, 0.72).

### Predicting DENV FOI and seroprevalence at 9 years of age across Timor-Leste

**Fig. 5:**
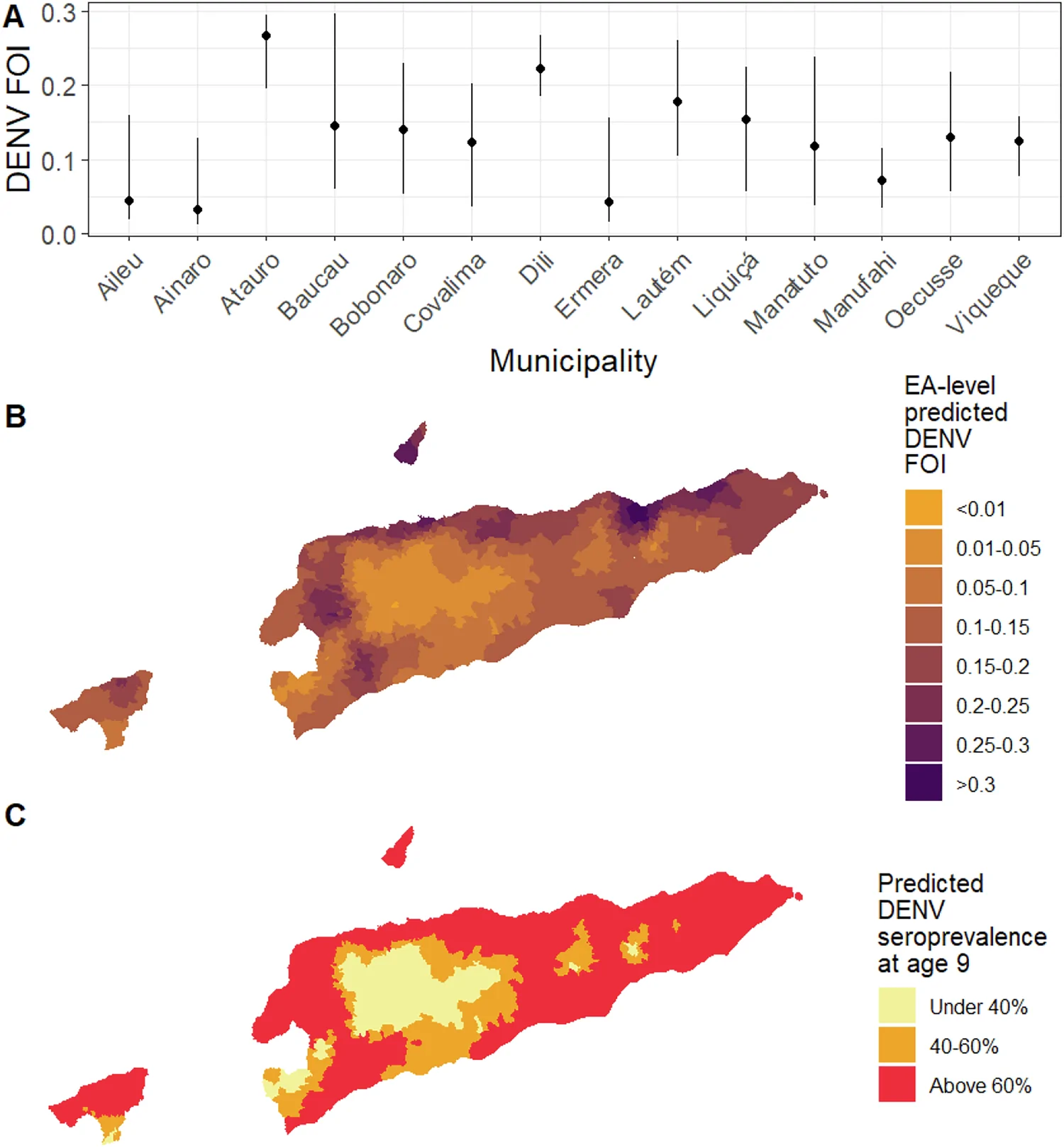
Predicted dengue virus (DENV) force of infection (FOI) and seroprevalence at 9 years of age across the 2350 enumeration areas (EAs) of Timor-Leste. **A** Median (point) and 95% quantile range (error bar) of the predicted EA-level dengue FOI grouped by municipality (number of EAs in each municipality: Aileu 113, Ainaro 130, Atauro 19, Baucau 267, Bobonaro 239, Covalima 153, Dili 296, Ermera 273, Lautém 156, Liquiçá 111, Manatuto 99, Manufahi 119, Oecusse 190 and Viqueque 185). **B** Predicted median total dengue FOI in EAs across Timor-Leste. **C** Predicted median seroprevalence at age 9 years in EAs across Timor-Leste.

The cross-validation analysis (out-of-sample testing where we fitted the model to 90% of the data and then compared its ability to accurately predict the remaining 10%) showed that the ensemble spatial model could reproduce the vast majority of the DENV FOI estimates and tended to underestimate the few DENV FOI at very high values, which were highly uncertain (Fig. S12). The spatial model predictions of DENV FOI across Timor-Leste show the highest FOI along the north coast, with hotspots visible in three urban centres (Dili, Baucau and Manatuto) as well as on Atauro Island (Figs. 5B and S13). When aggregated to the municipality level, the median predicted total FOI for Atauro and Dili were 0.27 (95% CrI: 0.19, 0.29) and 0.22 (95% CrI: 0.19, 0.27) respectively, corresponding to a median expected seroprevalence at 9 years of age of 91% and 86%. Lautém, Liquiçá and Baucau municipalities had median total FOI estimates greater than or equal to 0.15, suggesting an average seroprevalence at 9 years of over 74%. On the other hand, Aileu, Ainaro and Ermera had median total FOI estimates less than 0.05, corresponding to a median expected seroprevalence at 9 years of age of less than 36% (Figs. 5A, C and S13).

## Discussion

This nationally representative DENV seroprevalence survey in Timor-Leste is one of the few studies published to date investigating local variations in DENV seroprevalence and transmission at high spatial resolution. In general, FOI is high, exceeding 0.05 in most areas of Timor-Leste. Similarly, seroprevalence at 9 years is generally high (>60%). Nevertheless, at the fine scale, there are significant variations in FOI across the country and within municipalities. DENV transmission is identified in rural as well as urban areas, and is associated with climate and environmental variables. Several hotspots are identified where transmission is particularly high.

These findings are consistent with data from other countries in the Southeast Asian region where serological surveys have occurred. In 2014, Tan et al. undertook a study of 3194 children in 30 urban areas in Indonesia, which was the only previous Southeast Asian study to report geographically disaggregated data from five or more locations. DENV FOI varied from 0.043 (95% confidence interval, CI, 0.031–0.060) in Pulung, Jawa Timur, to 0.300 (95% CI 0.224–0.402) in Kendari, Sulawesi Tenggara, and seropositivity was associated with higher population density and ‘City’ (vs. ‘Regency’) administrative status^7,15^. Various other recent surveys have studied populations in Laos, Malaysia, Thailand and Vietnam, and have led to regional FOI estimates ranging between 0.001 (95% CrI: 0.000, 0.005)–0.219 (95% CrI: 0.154, 0.306), 0.010 (95% CrI: 0.008, 0.012)–0.059 (95% CrI: 0.052, 0.066), 0.070 (95% CrI: 0.060, 0.081)–0.136 (95% CrI: 0.127, 0.146) and 0.012 (95% CrI: 0.005, 0.029), in each country, respectively^15^.

This study identified transmission of DENV across many rural areas, which are traditionally considered less affected by DENV due to the ecology of *Aedes aegypti*, which is considered the main vector. Relative humidity was retained in the majority of the spatial models, which suggests that it is an important predictor of DENV FOI in Timor-Leste. Higher relative humidities were associated with lower DENV FOI, which is in line with the current understanding on the effect of atmospheric moisture levels impacting mosquito fitness and hydro-regulation at low and high extremes, and therefore arbovirus transmission^27^. For example, high humidity and temperature can reduce larval survival and egg-to-adult emergence through impacts on aquatic larval habitats^27^. Relative humidity, temperature and elevation are linked—for example, relative humidity is highest in the mountainous regions and lowest on the coast (Figs. S7 and S8). Therefore, observed associations between these variables and DENV FOI are consistent with high transmission in populated urban centres along the coast. The FOI estimates were highest in Dili municipality, which contains the largest and most densely populated city in Timor-Leste, and which historically reports most of the dengue cases in the country, and Atauro^2,21^. As well as being densely populated, urban centres typically serve as transportation hubs, have high rates of mobility, and contain human-made water storage containers in which *Aedes aegypti* mosquitoes can breed^28,29^.

Hotspots of transmission were identified in areas were case notification data are limited, including in some rural areas. Between 2005 and 2013, notified dengue cases were reported from Dili (which at the time included Atauro) (53.5%), Manatuto (16.7%), Baucau (14.2%) and Bobonaro (11.0%) municipalities, with the remaining municipalities reporting <2% of cases. At the village level, only 83 out of 442 locations reported any cases^2^. These data are consistent with the FOI estimates from the present study, which show hotspots of transmission in Atauro, Dili and Baucau. However, it also found high transmission intensity across locations in the North and East coasts and in the South-West of the country, where few dengue cases have been historically reported. This highlights a key advantage of using serological data to describe the infection burden of DENV and its distribution across the country, regardless of the sensitivity of case-based surveillance, including in remote communities where access to healthcare and diagnostic testing may be limited. Efforts to improve surveillance should be targeted in the locations identified as having a substantial DENV exposure as measured by the population seroprevalence and FOI. Future studies should compare the infection burden, as reconstructed from the FOI estimates generated in this study, to the number of cases reported from the same locations in Timor-Leste, thereby directly measuring the sensitivity of case-based surveillance and potentially assessing for age-related patterns in healthcare seeking and case-ascertainment.

The substantial number of geolocated FOI estimates generated in this study over a relatively small spatial scale contributes significantly to informing local and global efforts in DENV risk mapping, as it provides real-world evidence of the burden of infection and its variations across the country. It provides useful baseline data prior to the imminent deployment of Wolbachia-infected mosquitoes in Timor-Leste. Furthermore, following the latest WHO guidelines, the reconstructed seroprevalence at 9 years of age provides the data required by the national government to evaluate the introduction of the TAK-003 (and potentially other future) DENV vaccination programmes^26^. Future follow-up surveys targeted to appropriate age groups could be used to evaluate the impact of these interventions.

This study has some limitations. Whilst significant effort was made to ensure participants were representative of the Timorese population during survey design and implementation, the sampling frame was households enumerated during the 2015 national census, and it is possible that these census data were outdated when used in this survey approximately 6 years later. While Timor-Leste is a country with historically high numbers of internally displaced people, this has reduced significantly since it regained independence in 2002. Therefore, the effect of individuals being displaced (i.e. moving to new dwellings which were not in the study sampling frame) between 2015 and 2023 is considered to be small. Similarly, DENV FOI estimates were obtained according to the residence location of the study participants at the time of sample collection, and it is possible that individuals may have acquired the infection in another EA when travelling. This effect is also considered to be small, but can potentially be challenged in the future with the analysis of migration and mobility data. With regards to serological testing, a commercially available assay designed to detect previous non-serotype-specific DENV infections was chosen. Innovation in multiplexed assays may potentially allow to estimate serotype-specific FOIs in the future, beyond being a useful tool for simultaneously testing against multiple arboviruses and other pathogens using different antigens^18,20,30^. With regards to data analysis, our model estimates a time-constant average FOI, which does not capture seasonal and interannual heterogeneities in exposure. In highly endemic settings, time- or age-varying FOI estimates are best reconstructed from sequential cross-sectional seroprevalence surveys. An advantage of reconstructing time-constant transmission intensity estimates at the EA-level is the opportunity to compare the FOI across countries and globally. Whilst some EA-level FOI estimates have wide confidence intervals, we carried this uncertainty through into the environmental and spatial analyses. Our spatial modelling used averaged meteorological variables over the time period 2011–2022, to match the long-term temporal averaged nature of the DENV FOI estimates, which may not have captured spatial differences in extremes (e.g. in the minimum temperature), which have changed the most during the last decades. Finally, for some EAs, the size of the pixel (available at 1 km–5 km resolution) used to extract climate and environmental variables was larger than the size of the EAs, which may have introduced some approximations.

Despite these limitations, this study has several strengths. To the best of our knowledge, this is the largest seroprevalence study conducted so far in Southeast Asia in terms of its sample size and the number of geographic areas studied. It recruited individuals in randomly selected households in randomly selected EAs, resulting in a nationally-representative sample. It used hierarchical catalytical modelling, which accounted for the effects of household clustering effects and assay sensitivity/specificity, and examined a range of climate and environmental factors derived from satellite data covering the whole of the study area. This study provides unprecedented insights into the extent of dengue circulation across both rural and urban communities, robust FOI estimates, and gives insight into the drivers of DENV transmission across Timor-Leste.

In conclusion, this population-representative DENV serosurvey estimated the FOI at high spatial resolution across Timor-Leste. It found evidence of high DENV transmission intensity across most of the country, corresponding to >60% seroprevalence at 9 years of age, including spatially clustered and climate/environment-driven hotspots along the North and East coast and in urban areas. Given the concentration of case-based surveillance in urban centres and especially in Dili, the data presented in this study show that DENV infection is widespread across the island, including in rural areas. As such, this study can inform efforts to strengthen case-based surveillance and mosquito control interventions. This serosurvey provides the evidence needed to guide local decisions on the implementation and deployment of additional interventions, including DENV vaccines and the deployment of *Wolbachia*-carrying mosquitoes, beyond informing ongoing efforts to assess the local, regional and global burden of DENV infection.

## Methods

### Ethical considerations

This study complies with all relevant ethical regulations and received ethical approval from the Instituto Nacional da Saúde (INS) Research Ethics Committee, Timor-Leste (Reference: 875 MS-INS/DGE/IX/2021) and the Northern Territory Human Research Ethics Committee, Australia (Reference: 2021-4064). Adult participants were given verbal and written information about the purpose of the proposed study and its procedures and provided informed written consent. For children, verbal assent was sought, in addition to written consent from their parent or guardian.

### Study area

Timor-Leste consists of 13 municipalities, including Oecusse, which is an exclave within Indonesia and a Special Administrative Region (Fig. 1). Atauro Island was part of the Dili municipality at the time this study was conducted. During the Timor-Leste Population and Housing Census in 2015, the country was divided into 2350 enumeration areas (EAs) with an average of 89 households per EA^31^. The EA boundaries roughly correspond to the boundaries of ‘*aldeias*’, which can be considered similar to neighbourhoods (in urban areas) or hamlets/villages (in rural areas).

### Sampling strategy

The cross-sectional survey had a three-stage design, with random selection of EAs and households. Its design was informed by the World Health Organization Vaccination Coverage Cluster Surveys Reference Manual (2018)^32^. One-hundred-and-thirteen EAs were randomly selected from all EAs in the country, with probability proportionate to municipality population to ensure all municipalities were represented in the final dataset. Twenty-three households were then randomly selected from all households available within each EA. During fieldwork, which occurred between October 2021 and February 2023, households were located using GPS devices and bespoke maps (one created for each EA). They were visited up to three times, with all occupants aged over 1 year who were present at any of the visits being offered participation in the study. Those who were needle-phobic or had specific conditions putting them at risk during phlebotomy were excluded. Where the selected household was visited and found to be derelict, destroyed or no longer a household, the adjacent household could be selected as a replacement.

### Sample collection and analysis

Study nurses collected data (including age and gender) into questionnaires on electronic tablets and then took blood samples by phlebotomy from each participant. Target sample volume was 10 ml for adults (16+ years) and 5 ml for children (1–15 years). In adults and older children (6+ years), a 21–23 G needle and syringe was used. In younger children (1–5 years), a 23 G butterfly needle and syringe was used. Samples underwent centrifugation at 1500 RCF for 10 min and was then refrigerated at 2–8 °C in the field. Serum samples were transported to Laboratorio Nacional da Saude (LNS) in Dili, Timor-Leste, where they underwent serological analysis using the Panbio Indirect dengue IgG enzyme-linked immunosorbent assay (ELISA). This assay detects human IgG antibodies against any DENV and does not differentiate serotypes. This assay was chosen because it was designed for detecting past (as opposed to acute) DENV infections, has acceptable performance compared to the gold-standard plaque-reduction neutralisation test (PRNT) and has been used in serological surveillance studies previously^33–35^. Testing was performed in a singlet, in accordance with manufacturers’ instructions: cut-off values were calculated by multiplying the manufacturer’s batch calibration factor by the mean optical density (OD) of calibrators on each run. Index values (IVs) were then assigned to each sample by dividing the sample ODs by the cut-off value. Samples with IV > 1.10 were assigned positive, and those with IV < 0.9 were assigned negative. The appropriateness of this serological cut-off was verified visually (Fig. S5). In the main analysis, samples with IV between 0.9 and 1.1 (in the manufacturer’s suggested ‘equivocal range’) were also assigned negative; in a sensitivity analysis, we excluded the equivocal samples.

### Climate, environmental and population data

Mean maximum temperature (degrees Celsius), mean minimum temperature (degrees Celsius), precipitation accumulation (mm) and mean vapour pressure (kPa) for each month in the 11 years prior to the serosurvey (January 2011 to December 2022) were extracted from TerraClimate as 5 km resolution pixels^36,37^. Relative humidity was calculated from the temperature, and vapour pressure (see Supplementary Methods). Elevation above sea level data as 1 km-resolution pixels was extracted from WorldClim^38^. Mid infrared reflectance (MIR) and enhanced vegetation index (EVI) as 1 km-resolution pixels were downloaded for 2011–2022 in 8-day time intervals from NASA Moderate Resolution Imaging Spectroradiometer (MODIS) satellite data^39^. MIR is differentially reflected by vegetated, agricultural, and urban surfaces, and EVI is a calculated measure (derived from reflectance in the red, near infrared, and blue wavebands) which shows the density of plant growth. Annual population data between 2011 and 2021 was downloaded from Landscan^TM^^40^. For each of these variables, mean values were calculated for each pixel from the monthly observations between January 2011 and December 2022. This was achieved using the *raster, terra, and tidyterra* R packages (Figs. S6–S8)^41–43^. Meteorological, environmental, and population mean pixel values were then interpolated onto the EAs in Timor-Leste. For each EA, we calculated the weighted mean of the pixels which intersected with the EA boundaries given in a national shapefile. For example, for an EA entirely covered by one pixel (as was often the case for smaller EAs with the 5 km-resolution meteorological pixels (Fig. S6)), the value of the pixel was taken. For an EA intersected by several pixels, each pixel was weighted by the fraction of the EA it intersected, and the weighted average of the pixel values was taken. The calculation was performed using the *exactextract* package in R^44^. The collinearity between EA-level climate, environmental and population variables were assessed^45^.

### Sero-catalytic model

Previously published methods for estimating DENV FOI from age-stratified seroprevalence data were utilised to define a hierarchical Bayesian catalytic model (Model 1) to estimate the village (EA) age- and time-constant FOI from serological test data^4,6,14,15^.

We assumed endemic transmission of DENV in all analysed EAs. The model represents the FOI on the logit scale, with deviation from the national FOI at both the village (total of 102 villages) and household levels (1755 households) to capture spatial heterogeneity in transmission. The model accounts both for the nested structure of individuals within households and households within EAs and assumes imperfect test sensitivity and specificity.

Let *N* denote the number of individuals, *H* the number of households, and *V* the number of EAs. Let ${y}_{i}$ denote the observed serological test result for individual *i* (${y}_{i}=1$ if positive, 0 if negative). Each individual *i* belongs to household ${h}_{i}\in \{1,...,H\}$, and each household belongs to an EA ${v}_{{h}_{i}}\in \{1,...,V\}$. The age of individual *i* is reported as ${a}_{i}$.

The model assumes that the transmission follows a constant force of infection (${\lambda }_{i}$) for each individual, with hierarchical variation across EAs and households. The structure of the model is as follows.

For each village *v*:

1 $${{\rm{logit}}}\left({\lambda }_{v}\right)={\mu }_{v}={\mu }_{{{n}}}+{\delta }_{v}$$

where:

- ${\mu }_{{\mbox{n}}}$ is a national mean logit-FOI, taken from the Dengue FOI world map (DengueMap)^46^ estimate for Timor-Leste, providing an informative reference consistent with previous national-level spatial models.
- ${\mu }_{{\mbox{v}}}$ is the village level logit-FOI,
- ${\delta }_{v}{{\mathscr{\sim }}}{{\mathscr{N}}}(0,{\sigma }_{v})$ is a village-level deviation from the national mean, with standard deviation ${\sigma }_{v}$ (single SD shared across villages, estimated parameter).

In this Bayesian hierarchical framework, ${\mu }_{n}$ and ${\sigma }_{v}$ serve as hyperparameters that define the distribution from which the village-level parameters ${\mu }_{v}$ are drawn.

For each household *h*:

2 $${{\rm{logit}}}\left({\lambda }_{h}\right)={\mu }_{{v}_{(h)}}+{\delta }_{h}$$

where:

- ${\mu }_{{v}_{(h)}}$ is a village level logit-FOI of the village v to which household h belongs to.

- ${\delta }_{h}{{\mathscr{\sim }}}{{\mathscr{N}}}(0,{\sigma }_{h})$ is a household-level deviation from the village mean, with standard deviation ${\sigma }_{h}$ (single SD shared across households of all villages, estimated parameter).

For each individual i:

3 $${\lambda }_{i}={\lambda }_{{h}_{(i)}}$$

where the individual-level average yearly total FOI $\left({\lambda }_{i}\right)$ corresponds to the household-level FOI $\left({\lambda }_{{h}_{(i)}}\right)$ of the household *h* to which individual *i* belongs to.

The probability of having been exposed (seroprevalence) for individual *i* $\left({z}_{i}\right)$ is modelled as:

4 $${z}_{i}=1-\exp (-{\lambda }_{i}{a}_{i})$$

The observed individual-level probability of testing positive (${p}_{i}$) is calculated accounting for imperfect test sensitivity *se* and specificity sp as follows:

5 $${p}_{i}={se} \cdot \,{z}_{i}+\left(1-{sp}\right)\cdot\left(1-{z}_{i}\right)$$

The likelihood of the model is:

6 $${y}_{i} \sim {{\rm{Bernoulli}}}\left({p}_{i}\right)$$

Informative prior distributions were used to reflect plausible epidemiological ranges as represented in Table S2. Prior mean values for the test sensitivity and specificity were taken from Lopez et al.^34^.

A sensitivity analysis on the prior distributions of the test sensitivity and specificity was performed (using ${se} \sim {{\mathscr{N}}}({0.77,0.05})$ and ${sp} \sim {{\mathscr{N}}}({0.93,0.05})\left)\right.$.

The hierarchical model was implemented in a Bayesian framework in the *Rstan* software, and we used Hamiltonian Monte Carlo for parameter inference^47^. Three chains were run with 15,000 iterations with a burn-in period of 5000 iterations, and convergence was assessed visually and using the Rhat and the effective sample size statistics. The median and 95% credible interval (CrI) of the estimated parameters were extracted.

### Assessing drivers of DENV transmission

The posterior estimates of the 102 EA-level FOIs obtained from the sero-catalytic model were randomly sampled 100 times and log-transformed, and spatial regression models were fitted to a random 90% of each of these samples (with the remaining 10% of the FOI estimates withheld for out-of-sample cross-validation). Spatial regression models were fitted using the stochastic partial differential equations (SPDE) approach in *R-INLA*^48,49^.

The FOI across Timor-Leste was assumed to be a spatially correlated continuous process, which can be represented as a discrete process (μ, herein called spatial field). The assumption of spatial correlation was confirmed in initial checks (see Supplementary Methods). The spatial field was built on a triangulated mesh covering the coordinates of all the EA centroids (*n* = 2350) (see Supplementary Methods, Table S7 and Fig. S9) and was combined with an intercept (α) and j variables of interest (${X}_{j}$, with ${\beta }_{j}$ regression coefficient), to model the log-transformed total FOI for each of the 100 samples (Eq. 5). The nine independent variables (Table S6 and Figs. S7, S8) were standardised and added to the spatial models using the *INLAutils* package^50^, which uses forward selection to build the best-fitting model.

7 $${\log }(\lambda _{l})=\alpha+{\mu }_{l}+{\beta }_{1}{X}_{1}+\ldots+{\beta }_{j}{X}_{j}$$

### Predicting DENV FOI and seroprevalence at 9 years of age across Timor-Leste

The EA-level median and 95% CrI from the joint 100 posterior FOI spatial model estimates were extracted (*n* = 2350 EAs). Estimates of the expected seroprevalence at 9 years of age for each EA were generated using Eq. 6, and the median and 95% CrI of the joint 100 posterior estimates were extracted.

8 $${\varphi }_{l,9}=1-{e}^{-9{\lambda }_{l}}$$

The median predicted DENV FOI and the median predicted seroprevalence at 9 years of age (which is a measure used in WHO recommendations for vaccine suitability^26^) for each EA were mapped using the *sf* package to visualise the hotspots of transmission. The probabilities of the median seroprevalence at age 9 years exceeding 40% and 60% were estimated. The median, 2.5 and 97.5% quantiles of the EA-level FOI estimates within a municipality were calculated to estimate the municipality-level FOI and its uncertainty.

### Reporting summary

Further information on research design is available in the Nature Portfolio Reporting Summary linked to this article.

## Supplementary information

Supplementary Information

Reporting Summary

Transparent Peer Review File

## Data Availability

Study data are owned by the Instituto Nacional de Saúde Pública, Timor-Leste Ministry of Health. The data used in this analysis can be found in the following GitHub and Zenodo repositories: https://github.com/mrc-ide/Dengue_TimorLeste and https://zenodo.org/records/19495698. https://doi.org/10.5281/zenodo.19495655.

## Code availability

Code to reproduce the sero-catalytic and spatial modelling analyses can be found in the following GitHub and Zenodo repositories: https://github.com/mrc-ide/Dengue_TimorLeste and https://zenodo.org/records/19495698. https://doi.org/10.5281/zenodo.19495655.
