# Supplementary material for: Sero-catalytic and spatial modeling of dengue virus force of infection in Timor-Leste
Source: Nat Commun. 2026 Sep 15;17:10815. doi: 10.1038/s41467-026-76716-9 (PMC13669370; doi:10.1038/s41467-026-76716-9)
Supplement: Supplementary file 1 [file 41467_2026_76716_MOESM2_ESM.pdf]

nature portfolio

Corresponding author(s): Dr Paul Arkell

Last updated by author(s): May 8, 2026

Reporting Summary

Nature Portfolio wishes to improve the reproducibility of the work that we publish. This form provides structure for consistency and transparency in reporting. For further information on Nature Portfolio policies, see our Editorial Policies and the Editorial Policy Checklist.

Statistics

For all statistical analyses, confirm that the following items are present in the figure legend, table legend, main text, or Methods section.

n/a	Confirmed	
☒	☐	The exact sample size (*n*) for each experimental group/condition, given as a discrete number and unit of measurement
☒	☐	A statement on whether measurements were taken from distinct samples or whether the same sample was measured repeatedly
☐	☒	The statistical test(s) used AND whether they are one- or two-sided<br>*Only common tests should be described solely by name; describe more complex techniques in the Methods section.*
☐	☒	A description of all covariates tested
☐	☒	A description of any assumptions or corrections, such as tests of normality and adjustment for multiple comparisons
☐	☒	A full description of the statistical parameters including central tendency (e.g. means) or other basic estimates (e.g. regression coefficient) AND variation (e.g. standard deviation) or associated estimates of uncertainty (e.g. confidence intervals)
☒	☐	For null hypothesis testing, the test statistic (e.g. *F*, *t*, *r*) with confidence intervals, effect sizes, degrees of freedom and *P* value noted<br>*Give P values as exact values whenever suitable.*
☐	☒	For Bayesian analysis, information on the choice of priors and Markov chain Monte Carlo settings
☐	☒	For hierarchical and complex designs, identification of the appropriate level for tests and full reporting of outcomes
☒	☐	Estimates of effect sizes (e.g. Cohen's *d*, Pearson's *r*), indicating how they were calculated

Our web collection on statistics for biologists contains articles on many of the points above.

Software and code

Policy information about availability of computer code

Data collection	During fieldwork, demographic data were collected from participants into questionnaires on electronic tablets using redcap (version)
Data analysis	Climate, environmental and population data:<br>The mean values per pixel over the timeseries were calculated and visualised using the raster (version 3.6-30), terra (version 1.7-83), and tidyterra (version 0.6.1) packages in R<br><br>Sero-catalytic modelling:<br>Models were implemented in a Bayesian framework in Rstan (version 2.32.6) software<br><br>Spatial modelling:<br>Regression models were fitted using the stochastic partial differential equations (SPDE) approach in R-INLA (version 24.06.27).<br>Independent variables were standardised and added to the spatial models using the INLAutils (version 0.0.6) package in R.<br>Predicted dengue FOIs were mapped using the sf (version 1.0-19) package in R.

For manuscripts utilizing custom algorithms or software that are central to the research but not yet described in published literature, software must be made available to editors and reviewers. We strongly encourage code deposition in a community repository (e.g. GitHub). See the Nature Portfolio guidelines for submitting code & software for further information.

Data

Policy information about availability of data

All manuscripts must include a data availability statement. This statement should provide the following information, where applicable:

- Accession codes, unique identifiers, or web links for publicly available datasets
- A description of any restrictions on data availability
- For clinical datasets or third party data, please ensure that the statement adheres to our policy

Study data are owned by Instituto Nacional de Saúde Pública, Timor-Leste Ministry of Health. The data used in this analysis can be found in the following GitHub and Zenodo repositories: https://github.com/mrc-ide/Dengue_TimorLeste and https://zenodo.org/records/19495698.

Code to reproduce the sero-catalytic and spatial modelling analyses can be found in the following GitHub and Zenodo repositories: https://github.com/mrc-ide/Dengue_TimorLeste and https://zenodo.org/records/19495698..

Research involving human participants, their data, or biological material

Policy information about studies with human participants or human data. See also policy information about sex, gender (identity/presentation), and sexual orientation and race, ethnicity and racism.

Reporting on sex and gender	Participation from each municipality and dengue IgG seroprevalence rates are reported by gender.
Reporting on race, ethnicity, or other socially relevant groupings	Race and ethnicity of participants were not collected and are not reported in this manuscript.
Population characteristics	In this survey participants were randomly selected from census data and are therefore considered representative of the Timorese population. Age, gender, rural vs. urban household and dengue IgG seroprevalence are reported.
Recruitment	In this survey participants were randomly selected from census data and are therefore considered representative of the Timorese population.
Ethics oversight	This study received ethical approval from the Instituto Nacional da Saúde (INS) Research Ethics Committee, Timor-Leste (Reference: 875 MS-INS/DGE/IX/2021) and the Northern Territory Human Research Ethics Committee, Australia (Reference: 2021-4064).

Note that full information on the approval of the study protocol must also be provided in the manuscript.

Field-specific reporting

Please select the one below that is the best fit for your research. If you are not sure, read the appropriate sections before making your selection.

[x] Life sciences [ ] Behavioural & social sciences [ ] Ecological, evolutionary & environmental sciences

For a reference copy of the document with all sections, see nature.com/documents/nr-reporting-summary-flat.pdf

Life sciences study design

All studies must disclose on these points even when the disclosure is negative.

Sample size	The sample size calculation is described in a published protocol paper: https://bmjopen.bmj.com/content/13/5/e071381.long.<br><br>In brief, the strategy was designed to fulfil a target sample size such that enough individuals from each of five key age strata (1–4, 5–14, 15–24, 25–40, and 41+ years) would be included. The calculation was based upon an expected seroprevalence of 50%, a required precision of ±6% for the 95% conf idence interval (CI), and an overall design effect of 4. It provided a target sample size for each stratum of 1120 individuals (total sample size 5600 individuals).
Data exclusions	No data were excluded
Replication	The design of this study meant that findings were not replicated
Randomization	This was an observational study and participants were not allocated into groups. No randomization occurred.
Blinding	This was an observational study and participants were not allocated into groups. No blinding occurred.

Reporting for specific materials, systems and methods

We require information from authors about some types of materials, experimental systems and methods used in many studies. Here, indicate whether each material, system or method listed is relevant to your study. If you are not sure if a list item applies to your research, read the appropriate section before selecting a response.

Materials & experimental systems

n/a	Involved in the study
☒	☐ Antibodies
☒	☐ Eukaryotic cell lines
☒	☐ Palaeontology and archaeology
☒	☐ Animals and other organisms
☒	☐ Clinical data
☒	☐ Dual use research of concern
☒	☐ Plants

Methods

n/a	Involved in the study
☒	☐ ChIP-seq
☒	☐ Flow cytometry
☒	☐ MRI-based neuroimaging

Plants

Seed stocks	*Report on the source of all seed stocks or other plant material used. If applicable, state the seed stock centre and catalogue number. If plant specimens were collected from the field, describe the collection location, date and sampling procedures.*
Novel plant genotypes	*Describe the methods by which all novel plant genotypes were produced. This includes those generated by transgenic approaches, gene editing, chemical/radiation-based mutagenesis and hybridization. For transgenic lines, describe the transformation method, the number of independent lines analyzed and the generation upon which experiments were performed. For gene-edited lines, describe the editor used, the endogenous sequence targeted for editing, the targeting guide RNA sequence (if applicable) and how the editor was applied.*
Authentication	*Describe any authentication procedures for each seed stock used or novel genotype generated. Describe any experiments used to assess the effect of a mutation and, where applicable, how potential secondary effects (e.g. second site T-DNA insertions, mosiacism, off-target gene editing) were examined.*
