# Supplementary material for: Sero-catalytic and spatial modeling of dengue virus force of infection in Timor-Leste
Source: Nat Commun. 2026 Sep 15;17:10815. doi: 10.1038/s41467-026-76716-9 (PMC13669370; doi:10.1038/s41467-026-76716-9)
Supplement: Supplementary file 2 [file 41467_2026_76716_MOESM3_ESM.pdf]

nature portfolio

Peer Review File

Sero-catalytic and spatial modeling of dengue force of infection in Timor-Leste

Corresponding Author: Dr Paul Arkell

This file contains all reviewer reports in order by version, followed by all author rebuttals in order by version.

Version 0:

Reviewer comments:

Reviewer #1

(Remarks to the Author)
In this manuscript, the authors present a nationwide serosurvey to estimate the force of infection (FOI) for dengue virus (DENV) across diverse ecological areas in Timor-Leste, leveraging random selection from census data and environmental/demographic modeling to infer transmission risk in under-sampled regions. The study aims to address a critical gap in understanding spatial heterogeneity of DENV transmission, which directly influences vaccine deployment and vector control strategies. Strengths include the ambitious scope, focus on both urban and remote areas, and attempts to integrate multiple data streams to overcome surveillance limitations. However, a number of methodological and analytical concerns—including plausibility of FOI estimates, sampling bias, and serological assay limitations—limit the current utility of results for policy and intervention planning.

MAJOR COMMENTS
Estimation of FOI:
Some FOI estimates, particularly in Dili, are implausibly high (up to 68%). This raises concerns about both methodological appropriateness and bias. Please clarify whether an average (constant) FOI was assumed, particularly in light of a recent large epidemic (in 2022). Provide a rationale for using this approach instead of time-varying or age-varying alternatives, and discuss possible impacts of this assumption in your discussion.

Sampling and Urban-Rural Bias:
The sampling method appears to preferentially include individuals from urban areas, requiring extrapolation for rural settings. Please discuss how this may affect your ability to reliably estimate FOI in rural areas. Also, comment on the reliability of inferring associations between DENV exposure and environmental/climatological variables based primarily on urban data, as these associations may differ between environments.

Geographic Assignment and Exposure Source:
Fine-scale FOI estimates assigned to the EA level assume limited mobility between EAs and presume infection occurred within the individual's home EA. In reality, infections may have occurred elsewhere, which is not discussed and could bias results.

Enrollment Bias and Census Limitations:
Please provide data or a table showing, among those randomly selected from the census, how many opted to participate. With seroprevalence rates in some EAs exceeding 95%, enrollment bias is a concern—particularly since regions with high IgG positivity correspond to areas with recent outbreaks (e.g., Dili in 2022). Clarify recruitment and enrollment procedures. Further, discuss any limitations of census-based sampling in a country with historically high numbers of internally displaced people; are these groups represented? Are other distinct risk groups potentially missing?

Assay Sensitivity and Misclassification:
The Panbio Indirect dengue IgG ELISA is known to have poor sensitivity, especially for primary infections (possibly as low as 33%: https://www.globalpointofcare.abbott/th/en/product-details/panbio-dengue-igg-indirect-elisa.html). This can significantly impact your estimates, and assuming 100% sensitivity is overly optimistic. Please comment on assay limitations, discuss the likely impact on your analyses, and consider including sensitivity analyses or alternative methods that account for assay performance. Note also that any implausibly high FOI estimates for some EAs, especially in Dili,

would likely be even higher if the assay's limited sensitivity were fully considered.

Interpretation of FOI Variability:
Please discuss the reasonableness of wide variation in FOI estimates at the EA level, including within the same sub-district (e.g., 11% to 65% in Dom Aleixo, Dili). Would you consider this a successful approach at the EA level, or is it more reliable at the municipal level? As written, the wide variability in EA-level estimates (and implausibly high values) suggests they may not be ready to support or evaluate interventions—perhaps they are more appropriate for use at the municipal level, after addressing other concerns about bias and estimation methods.

Significance of Work:
Above concerns aside, this study addresses an important and novel knowledge gap for dengue control: How much heterogeneity exists in endemic countries, on what scale, and what factors drive it? The approach to infer dengue risk for remote areas with limited access to care and testing is also valuable for identifying future surveillance and intervention priorities.

MINOR COMMENTS
• Use 'DENV' instead of 'dengue' throughout, except when referring to clinical disease.
• In the Introduction, reference FOI ranges (by serotype and overall) from prior publications when discussing previous estimates.
• Move mention of imminent Wolbachia deployment in Timor-Leste earlier in the Introduction, rather than much later in the manuscript.
• In the results section ('Predicting dengue FOI and seroprevalence at 9 years of age…'), define 'out-of-sample testing' or use clearer phrasing for lay readers.
• In the Discussion (paragraph 1), clarify that rural areas are not necessarily less affected by dengue. You may note that robust methods may help identify high-risk rural areas even with limited case detection, however.
• When discussing the impact of relative humidity (Discussion, paragraph 3), please reference additional literature on associations between humidity and mosquito fitness. A broader review would be helpful, even if brief.
• When discussing associations between environmental/climate variables and FOI, elevation is mentioned as a potentially important factor—consider measuring it directly and including it in your model.
• In Discussion, paragraph 4 ("Between 2005 to 2013, cases were reported…"), explain why only data through 2013 is used. More current and comprehensive surveillance data would further support your evaluation and discussion, acknowledging the challenges of surveillance bias and underreporting.
• In Discussion, paragraph 6 ("Innovation in multiplexed microsphere immune assays may potentially allow estimation of serotype-specific FOIs"), broaden this reference to highlight that multiple assay platforms and approaches are in development for serotype-specific FOI estimation (e.g., "innovation in multiplexed assay approaches may…").

(Remarks on code availability)

Reviewer #2

(Remarks to the Author)
The authors present the results of a nationally represented dengue seroprevalence survey and calculated the force-of-infection and identified significant drivers.

Introduction
- Well written, includes the needed content to justify and provided context to their study and approach to analysis

Methods
- A bit more information should be included in the sampling approach. When EAs were first visited, were all of the households enumerated (and included information on HHs with children/infants > 1) and a simple random selection was conducted based on this list? No stratified SRS of EAs? Why not?
- The blood draw was conducted during the household visit by a study nurse?
- Sample size calculation for age stratified clustered data?
- Lab test – equivocal samples – were they retested?
- Missing clinical data, how was this handled?
- Climate and environmental data - what was the imputation approach for missing data? how much variation is there seasonally with the different climatic/environmental variables? And from year to year? Unclear, even after reading the supplement, how the time series data were converted into cross-sectional data. This information is needed to understand this approach in terms of limitations/interpretation etc.
- Collinearity in the variables in the spatial regression model, was this assessed?
- How were the various models assumptions checked?

Results
- Remove the mention of insignificant associations (EVI + dengue FOI)
- Figures:
o It would be more intuitive to have IgG + as red vs blue
o Figure 2a – what is the x-axis? Time?

o Figure 3 – here IgG+ HH is red. I don't find this figure very informative. Is the another way to illustrate this concept or replace with text?
o How we interpret FOIs in Figure 4? For example, a FOI = 1 means total per capita risk?
o Figure 5 a– given the range of FOI in Figure 4, wouldn't it make more sense to have range of the FOI better represented ? Figure 5c – unclear, is this for age 9 as well?

Discussion
- Well written and structured.
- It would be helpful to further contextualize these results by comparing to other dengue endemic countries (the countries mentioned in the introduction).

(Remarks on code availability)

Version 1:

Reviewer comments:

Reviewer #1

(Remarks to the Author)
In this revised manuscript, the authors present a nationwide serosurvey to estimate the force of infection (FOI) for dengue virus (DENV) across diverse ecological areas in Timor-Leste, using random selection from census data and environmental/demographic modeling to infer transmission risk in under-sampled regions. The study addresses an important gap in understanding spatial heterogeneity of DENV transmission, with direct relevance for vaccine deployment and vector control. The revision is substantially stronger: previous concerns about FOI estimation, sampling and participation (including rural/urban representation), assay sensitivity and specificity, and the treatment of environmental covariates and spatial heterogeneity have been thoughtfully and transparently addressed. Remaining caveats relate primarily to cautious interpretation of the very highest EA-level FOI estimates, which are now clearly presented in the context of wide credible intervals, alongside more stable municipality-level estimates, and discussed as such in the manuscript. No major concerns remain.

(Remarks on code availability)

Reviewer #2

(Remarks to the Author)
Thank you to the authors for their revision. The vast majority of my comments have been adequately addressed although there remain some outstanding issues:

Methods:
- three-stage cluster-random survey should be instead three stage survey design with random selection of EAs and households.
- children as young as 1 yrs old, had a full blood draw? And was it a full blood draw or only serum using specialized tubes? Volume taken? More information is needed

Discussion
- the first paragraph needs to summarize the key findings/take home messages and remove the comparison to other studies - this should go elsewhere in the discussion

(Remarks on code availability)

Open Access This Peer Review File is licensed under a Creative Commons Attribution 4.0 International License, which permits use, sharing, adaptation, distribution and reproduction in any medium or format, as long as you give appropriate credit to the original author(s) and the source, provide a link to the Creative Commons license, and indicate if changes were made.
In cases where reviewers are anonymous, credit should be given to 'Anonymous Referee' and the source.
The images or other third party material in this Peer Review File are included in the article's Creative Commons license, unless indicated otherwise in a credit line to the material. If material is not included in the article's Creative Commons license and your intended use is not permitted by statutory regulation or exceeds the permitted use, you will need to obtain permission directly from the copyright holder.
To view a copy of this license, visit https://creativecommons.org/licenses/by/4.0/

REVIEWER COMMENTS

Reviewer #1 (Remarks to the Author):

In this manuscript, the authors present a nationwide serosurvey to estimate the force of infection (FOI) for dengue virus (DENV) across diverse ecological areas in Timor-Leste, leveraging random selection from census data and environmental/demographic modelling to infer transmission risk in under-sampled regions. The study aims to address a critical gap in understanding spatial heterogeneity of DENV transmission, which directly influences vaccine deployment and vector control strategies. Strengths include the ambitious scope, focus on both urban and remote areas, and attempts to integrate multiple data streams to overcome surveillance limitations. However, a number of methodological and analytical concerns—including plausibility of FOI estimates, sampling bias, and serological assay limitations—limit the current utility of results for policy and intervention planning.

We thank the reviewer for the positive review and appreciate the constructive feedback provided. As explained in greater detail below, we have implemented new hierarchical methods to estimate the FOI – thus overcoming the concerns about the small samples sizes and strengthening the methodological approach used in the manuscript. Even with the new revised methods, a few FOI estimates remain higher than previously observed. We attribute this to the fine scale analysis and epidemiological nature of dengue transmission in Timor Leste, which is a key contribution of this study to the existing literature.

MAJOR COMMENTS

Estimation of FOI:

Some FOI estimates, particularly in Dili, are implausibly high (up to 68%). This raises concerns about both methodological appropriateness and bias. Please clarify whether an average (constant) FOI was assumed, particularly in light of a recent large epidemic (in 2022). Provide a rationale for using this approach instead of time-varying or age-varying alternatives, and discuss possible impacts of this assumption in your discussion.

We appreciate the reviewer's comment and understand the concern regarding the apparently high FOI, particularly for some enumeration areas (EAs). Indeed, the high FOI estimates in these locations largely reflect the high seroprevalence observed in the data given the fine spatial resolution of our analysis. Using the methods implemented in the original submission, we estimated the FOI from the results observed in each EA considered independently, which may pose some limitations given there may be limited power in the data due to relatively small sample sizes. We have therefore substantially revised the methods used to estimate the FOI, and in the revised paper we estimate EA-level FOIs by fitting the island-wide data using a

hierarchical model, which allows us to estimate the household- and EA-level variations in the dengue FOI from a nationwide distribution. With the updated hierarchical model, the highest FOI estimates do not exceed 0.6. Whilst we appreciate that such high FOI estimates have not been published before, these appropriately capture the observed age trends in seroprevalence and unveil the extent of dengue transmission across the island.

The seroprevalence data were collected between October 2021 and February 2023. We have assumed an average, constant FOI per EA because the seroprevalence data show a clear endemic transmission pattern and in fact, a constant-in-time dengue FOI captures the observed trends accurately (as shown in Figure S13). Models assuming time-varying or age-varying EA level FOI estimates from a single cross-sectional survey could face identifiability issues given the substantial number of additional parameters, and the large number of EAs (102). On the other hand, there is epidemiological value in characterising the extent of FOI variation at the EA and household level, and constant-in-time FOI estimates for endemic settings are valuable as they allow estimation of key outcomes such as the seroprevalence at 9 years of age, which is critical for evaluating the potential local implementation of the Takeda vaccine according to WHO guidelines. The current global dengue transmission and burden maps are built on constant-in-time dengue FOIs, and the estimates presented in this work can contribute to similar and ongoing global burden mapping efforts upon publication.

However, we acknowledge that there can be year on year variations in FOI also in endemic settings, and we have added a discussion point in the manuscript to highlight the limitations and advantages of this assumption.

Sampling and Urban-Rural Bias:
The sampling method appears to preferentially include individuals from urban areas, requiring extrapolation for rural settings. Please discuss how this may affect your ability to reliably estimate FOI in rural areas. Also, comment on the reliability of inferring associations between DENV exposure and environmental/climatological variables based primarily on urban data, as these associations may differ between environments.

We thank the reviewer for raising this important point. This study used the 2015 national census, which enumerated and geolocated all households in Timor-Leste, as its sampling frame. First, enumeration areas (EAs) were randomly selected from all EAs in the country. They were selected with probability proportionate to municipality population to ensure all municipalities were represented in the final dataset. Second, 23 households were randomly selected from all households available in each selected EA. Finally, all eligible occupants in each selected household were offered participation. This three-stage cluster sample strategy, which was informed by the World Health Organization Vaccination Coverage Cluster Surveys Reference Manual (2018) intended to derive a nationally population-

representative sample and is a strength of our study when compared to many other serological surveys which take samples which may not be representative. It resulted in a sample which was 73% rural.

To address the Reviewer's concern, we have extended the hierarchical model to account for the effect of urban versus rural EAs on FOI, as shown in the sensitivity analysis (Model 2, Supplementary Information). We found that the urban/rural classifier was not significant, i.e. there was no statistically significant difference in the FOI of rural vs urban EAs having allowed for EA-level variability.

Geographic Assignment and Exposure Source:
Fine-scale FOI estimates assigned to the EA level assume limited mobility between EAs and presume infection occurred within the individual's home EA. In reality, infections may have occurred elsewhere, which is not discussed and could bias results.

In the revised model, we fit all islandwide seroprevalence data jointly, whilst estimating the effect of the household structure (without having conditioned on the seropositivity of the household members) and of EA-level variability. The model does not make any explicit assumption on the location where infection occurred, though individuals are assigned to a residence location and a household. We agree that the EA-level FOI estimates are reconstructed from the EA-level seroprevalence, and that potentially individuals living in a specific EA may have acquired the infection in another EA. This is a standard assumption used when analysing seroprevelance data with catalytic models, which can potentially be tested in future work with mobility data. We have included this assumption and limitation in the Discussion.

Enrolment Bias and Census Limitations:
Please provide data or a table showing, among those randomly selected from the census, how many opted to participate.

This study used all households in Timor-Leste as the sampling frame (as opposed to individuals). These were randomly selected from 2015 census data, as described in an earlier answer above.   All participants in selected households were then eligible to participate. Further details of survey methods have been added to the methods section, and EA, household and individual participation rates for each municipality are detailed in Figure S10 & Table S3.

With seroprevalence rates in some EAs exceeding 95%, enrolment bias is a concern—particularly since regions with high IgG positivity correspond to areas with recent outbreaks (e.g., Dili in 2022). Clarify recruitment and enrolment procedures. Further, discuss any limitations of census-based sampling in a country with historically high numbers of internally displaced people; are these groups represented? Are other distinct risk groups potentially missing?

Thank you for these requests. Further details of survey methods and participation rates have been added to the methods section. Additionally, a flow diagram showing participation of households and household occupants is shown in Figure S10, and details of EA, household and individual participation rates for each municipality are given in Table S3. The median age of participants was 28 years (inter-quartile range, IQR 13-46 years) while the median age of non-participating household occupants (as described by the head-of-household during data collection) was 23 years (IQR 10-41 years). Therefore, there is some evidence that younger individuals were relatively under-sampled. . However, under or over sampling of different age groups is not expected to affect the central FOI estimates, because these are a function of age-stratified seroprevalence. We agree though that small sample sizes can affect the uncertainty of the FOI estimates.

With regards to displaced persons, we acknowledge that Timor-Leste is a country with historically high numbers of internally displaced people. However, this has reduced significantly since it regained independence in 2002. Therefore, the effect of individuals being displaced (i.e. moving to new dwellings which were not in the study sampling frame) between 2015 (census) and 2023 (survey) is considered to be small.   This consideration has been added to the discussion section.

Assay Sensitivity and Misclassification:
The Panbio Indirect dengue IgG ELISA is known to have poor sensitivity, especially for primary infections (possibly as low as 33%: https://www.globalpointofcare.abbott/th/en/product-details/panbio-dengue-igg-indirect-elisa.html). This can significantly impact your estimates, and assuming 100% sensitivity is overly optimistic. Please comment on assay limitations, discuss the likely impact on your analyses, and consider including sensitivity analyses or alternative methods that account for assay performance. Note also that any implausibly high FOI estimates for some EAs, especially in Dili, would likely be even higher if the assay's limited sensitivity were fully considered.

We agree with the Reviewer that the sensitivity and specificity of the Panbio Indirect dengue IgG ELISA can affect the FOI estimates. To account for this, we have now explicitly included the test sensitivity and specificity in the hierarchical model generating the FOI estimates. The prior distributions for sensitivity and specificity were centered on the values reported in https://pubmed.ncbi.nlm.nih.gov/33212030/ . These were chosen in preference to the values given in the manufacturers document (i.e. 33%) which are derived from testing samples from patients with dengue infections (i.e. during illness), including primary infections. In this situation there is usually a delay in the emergence of IgG which results in low sensitivity. In cross-sectional surveys aimed at detecting previous infections in non-febrile individuals, the sensitivity

of this assay is considerably higher. We conducted sensitivity analyses using both flatter and more informative prior standard deviations for these parameters. In both cases, the estimated sensitivity was 99% (95%CrI: 99, 100%) and the estimated specificity was 98% (95%CrI: 95, 100%), demonstrating that the FOI estimates are robust to reasonable variations in the assumed assay performance.

Interpretation of FOI Variability:
Please discuss the reasonableness of wide variation in FOI estimates at the EA level, including within the same sub-district (e.g., 11% to 65% in Dom Aleixo, Dili). Would you consider this a successful approach at the EA level, or is it more reliable at the municipal level? As written, the wide variability in EA-level estimates (and implausibly high values) suggests they may not be ready to support or evaluate interventions—perhaps they are more appropriate for use at the municipal level, after addressing other concerns about bias and estimation methods.

We appreciate the Reviewer's concerns on the original implementation of the methods. Having revised the methods, and having generated both EA- and municipality- (district-) level FOI estimates using a hierarchical modelling approach, we believe that a key strength of this analysis is in the demonstration of the steep age trends in seroprevalence and hence high FOIs that can be found when conducting analyses at high spatial resolution in high transmission settings. The uncertainty around some EA-level FOI estimates reflects their relatively small sample sizes at this level. We agree that municipality-level FOI estimates tend to be lower and less uncertain – this is fundamentally due to spatial averaging across locations and to the aggregation and hence increase of the sample sizes. By presenting both EA- and municipality-level FOI estimates, we provide to the readership an opportunity to choose the estimates that better suit future analyses.

Significance of Work:
Above concerns aside, this study addresses an important and novel knowledge gap for dengue control: How much heterogeneity exists in endemic countries, on what scale, and what factors drive it? The approach to infer dengue risk for remote areas with limited access to care and testing is also valuable for identifying future surveillance and intervention priorities.

Thank you for the positive feedback on the significance of the work.

MINOR COMMENTS
• Use 'DENV' instead of 'dengue' throughout, except when referring to clinical disease.

This change has been made

• In the Introduction, reference FOI ranges (by serotype and overall) from prior publications when discussing previous estimates.

This has been added.

• Move mention of imminent Wolbachia deployment in Timor-Leste earlier in the Introduction, rather than much later in the manuscript.

We have added imminent Wolbachia deployment to the abstract.

• In the results section ('Predicting dengue FOI and seroprevalence at 9 years of age…'), define 'out-of-sample testing' or use clearer phrasing for lay readers.

This section has been changed to "The cross-validation analysis (out-of-sample testing where we fitted the model to 90% of the data and then compared its ability to accurately predict the remaining 10%) showed that…"

• In the Discussion (paragraph 1), clarify that rural areas are not necessarily less affected by dengue. You may note that robust methods may help identify high-risk rural areas even with limited case detection, however.

Thank you for this comment. We have amended the discussion accordingly.

• When discussing the impact of relative humidity (Discussion, paragraph 3), please reference additional literature on associations between humidity and mosquito fitness. A broader review would be helpful, even if brief.

A broader review has been added.

• When discussing associations between environmental/climate variables and FOI, elevation is mentioned as a potentially important factor—consider measuring it directly and including it in your model.

Thank you for this comment. Elevation was already measured directly and included in the analysis, but was not chosen in the final models because other variables explained more of the variation.

• In Discussion, paragraph 4 ("Between 2005 to 2013, cases were reported…"), explain why only data through 2013 is used. More current and comprehensive surveillance data would further support your evaluation and discussion, acknowledging the challenges of surveillance bias and underreporting.

Thank you for this comment. Regrettably, recent, curated and geographically disaggregated case surveillance data from Timor-Leste were not available for use in this study. The lack of such data highlights the usefulness and impact of conducting serological surveillance studies such as the one presented in this manuscript. Nevertheless, we are working with partners in the Timor-Leste Ministry of Health to

collate the available surveillance data, which we hope to use in future work to compare the estimates obtained from case-based and serological surveillance. This would allow us to directly measure the sensitivity of case-based surveillance and to quantify any evidence of age-specific patterns in healthcare seeking and case-ascertainment.

• In Discussion, paragraph 6 ("Innovation in multiplexed microsphere immune assays may potentially allow estimation of serotype-specific FOIs"), broaden this reference to highlight that multiple assay platforms and approaches are in development for serotype-specific FOI estimation (e.g., "innovation in multiplexed assay approaches may…").

This change has been made.

Reviewer #2 (Remarks to the Author):

The authors present the results of a nationally represented dengue seroprevalence survey and calculated the force-of-infection and identified significant drivers.

Introduction
- Well written, includes the needed content to justify and provided context to their study and approach to analysis

We thank the reviewer for the positive feedback.

Methods
- A bit more information should be included in the sampling approach. When EAs were first visited, were all of the households enumerated (and included information on HHs with children/infants > 1) and a simple random selection was conducted based on this list? No stratified SRS of EAs? Why not?

Thank you for this comment. We have now included more details of the sampling strategy and response rates in the methods section, Figure S10 and Table S3. The survey protocol has also been published elsewhere: https://pmc.ncbi.nlm.nih.gov/articles/PMC10201250/

Households in EAs were enumerated and geolocated during the 2015 national census. We therefore used this household census data as our study sampling frame. EAs were randomly selected with probability proportionate to municipality population, which ensured all municipalities were represented in the sample. Households were then randomly selected from all enumerated households. This was done before EAs were visited, so that bespoke maps could be created and fieldwork routes between households (often on foot) could be planned in advance of each trip. Finally all individuals >1yr at each household were eligible.

- The blood draw was conducted during the household visit by a study nurse?

Yes this is correct. We have added this detail to the manuscript.

- Sample size calculation for age stratified clustered data?

Details of the sample size calculation for this survey, which had the primary aim of investigating vaccine-preventable disease seroprevalence (with secondary aim of measuring DENV FOI) was are given in the published protocol: https://pmc.ncbi.nlm.nih.gov/articles/PMC10201250/.

In brief: A sample size was calculated with reference to the WHO Reference Manual for Vaccination Coverage Cluster Surveys to give seroprevalence estimates for key vaccine-preventable diseases in five age groups: 1–4, 5–14, 15–24, 25–40 and 41+ years. An effective sample size was calculated of 280 for each stratum. This was based on an expected seroprevalence of 50% and a precision of ±6% for the 95% CI. Without local data on which to confidently base estimates of design effect, a conservative design effect of 4 was estimated, to ensure a sufficient sample to provide precise results. This was intended to account for various design effects, including the effect of household- and EA-level clustering. This provided a sample size for each strata of 1120 individuals, and a total survey sample size of 5600. A further calculation was made to estimate the number of households needed to reach this sample size, based on expected household size (5.7 occupants) and age spread from national Census data.

Sampling was not stratified by age. This was due to previous experience conducting surveys in Timor-Leste which suggested that it would not be practical and it would not be acceptable to several potential participants if occupants of particular ages were selected from households (while others were not). Instead, all occupants at selected households were offered participation, with the sample size being sufficient to reach target in the smallest age group (i.e. 1-4 years).

- Lab test – equivocal samples – were they retested?

Thank you for this question. A small minority of samples (57, 1.2%) had IV in the manufacturers' suggested 'equivocal range'. For the purposes of this study these were assigned as negative in the main analysis (and these details have been added to the methods section). However, we have now added a sensitivity analysis to asses the impact of assuming that the equivocal samples were negative; in the sensitivity analysis we removed the equivocal samples from the catalytic model fitting. The results are presented in the supplementary materials (Figure S4).

- Missing clinical data, how was this handled?

This study did not use any clinical data (i.e. data relating to participants becoming unwell with dengue infection). However, if the reviewer is asking about participants' demographic data then we can report that there was no missing data among any of the following variables: 'municipality', 'EA', 'household number', 'rural/urban' , 'age',

‘gender’. More detail around how these demographic details were collected by field staff have been added to the methods section.

- Climate and environmental data - what was the imputation approach for missing data?

Thank you for this question. Climate and environmental data were retrieved from open-source climate datasets (i.e. TerraClimate, WorldClim,MODIS, and Landscan^{TM}). These datasets include gridded climate data/estimates on a grid (i.e. pixels) at regular timepoints. We are aware that, during the production of these gridded data sets, imputation can be implemented. The imputation methods used are standardised, and users cannot distinguish the imputed climate data from the non-imputed data. Notably, the use of the resulting datasets is standard practice in climate research. Therefore, the inclusion of imputation for missing climate data was not required in this study.

how much variation is there seasonally with the different climatic/environmental variables? And from year to year? Unclear, even after reading the supplement, how the time series data were converted into cross-sectional data. This information is needed to understand this approach in terms of limitations/interpretation etc.

Thank you for this comment. Further details of how time series data were converted into cross-sectional data are now given in the methods section of the manuscript. Essentially, for each variable, we produced ‘mean values’ at each pixel. These were the sum of all values, divided by the number of values during the 11 year period for which we had data. Therefore, seasonal variation in climate and environmental variables was not captured, and our analyses did not assess whether the degree of seasonal variation affected dengue FOI – from a methodological perspective, this might be possible but is expected to be challenging and subject to identifiability issues given we had a single FOI estimate for each location. Whilst the range of values, quantile ranges, mean values during known wet/dry seasons, and parameters derived from fast Fourier transformations have been used in other studies, a more comprehensive exploration was not conducted in the light of the results seen with the current model and due to the inclusion of spatial and temporal effects which can capture unobserved variations. In conclusion, we appreciate the suggestion, but the assessment of the effects of changes in climate/environment would be better suited in a study estimating time-varying FOIs.

- Collinearity in the variables in the spatial regression model, was this assessed?

Collinearity between the variables in the spatial model was assessed using Pearson's correlation coefficient with a cut-off of >|0.6| indicating correlation. We have now included this and Figure S12 describing the collinearity can be found in the Supplementary Material.

- How were the various models assumptions checked?

Within the spatial regression model, the assumption of independence in the fixed covariate variables was upheld given the lack of collinearity in the variables (tested using Pearson's correlation coefficient, see response to point above). The spatial model also assumed that the DENV FOI is a continuous spatially-correlated process across Timor Leste. The INLA methodology allows us to represent this process using spatial random fields on a gridded mesh constructed using the EA coordinate locations. To check that the assumption of spatial autocorrelation was valid, we performed initial checks on the spatial auto-correlation within the EA-level DENV FOI estimates from the catalytic model and confirmed the presence of strong autocorrelation, thus justifying our choice of methodology. To check the autocorrelation we assigned each EA centroid its nearest six neighbours, based on distance between the coordinates, and computed the Moran I statistic for the DENV FOI at the coordinates using the *spdep* R package (Pebesma & Bivand, 2023); which indicates presence of spatial autocorrelation when the values are positive with significant p-values (Moran I statistic = 0.27. p-value = 1.585e-08).

Results
- Remove the mention of insignificant associations (EVI + dengue FOI)

We thank the reviewer for this suggestion. We believe it is important to mention the EVI variable because it was chosen in many of the model samples, however we have now adapted the sentence to make it shorter and state clearly that EVI was not a significant association in the ensemble model (the combined joint posteriors of all the model samples).

- Figures:
o It would be more intuitive to have IgG + as red vs blue

Figure 2 has been changed accordingly.

o Figure 2a – what is the x-axis? Time?

Thank you, the x-axis is age. This has been clarified in the Figure 2 caption.

o Figure 3 – here IgG+ HH is red. I don't find this figure very informative. Is the another way to illustrate this concept or replace with text?

This figure has now been removed, we have now 4 main figures

o How we interpret FOIs in Figure 4? For example, a FOI = 1 means total per capita risk?

FOI is the annual per-capita risk of infection for a susceptible person. This clarification has been added to Figure 3 (previously Figure 4), and a scale has been added to the x-axis (which was previously missing).

o Figure 5 a– given the range of FOI in Figure 4, wouldn't it make more sense to have range of the FOI better represented ?

With the error bars, we did not show the range in Figure 4 – we showed the 95% CrI. For this reason, there was consistency between Figure 4 and Figure 5 (now Figure 3 and Figure 4). We have provided the definition of the uncertainty as represented by the error bars in the caption of both Figures.

Figure 5c – unclear, is this for age 9 as well?

We agree that the original Figure 5 was potentially confusing. We have therefore moved the municipality FOI estimates to the top (A), followed by the EA-level FOI estimates (B) and EA-level estimates of seroprevalence at 9 years old (C). Please note this comment and response now relates to Figure 4.

Discussion
- Well written and structured.
- It would be helpful to further contextualize these results by comparing to other dengue endemic countries (the countries mentioned in the introduction).

Thank you, our results have now been compared to results from all other surveys which have occured in Southeast Asia.

REVIEWERS' COMMENTS

Reviewer #1 (Remarks to the Author):

In this revised manuscript, the authors present a nationwide serosurvey to estimate the force of infection (FOI) for dengue virus (DENV) across diverse ecological areas in Timor-Leste, using random selection from census data and environmental/demographic modeling to infer transmission risk in under-sampled regions. The study addresses an important gap in understanding spatial heterogeneity of DENV transmission, with direct relevance for vaccine deployment and vector control. The revision is substantially stronger: previous concerns about FOI estimation, sampling and participation (including rural/urban representation), assay sensitivity and specificity, and the treatment of environmental covariates and spatial heterogeneity have been thoughtfully and transparently addressed. Remaining caveats relate primarily to cautious interpretation of the very highest EA-level FOI estimates, which are now clearly presented in the context of wide credible intervals, alongside more stable municipality-level estimates, and discussed as such in the manuscript. No major concerns remain.

Thank you for your thoughtful review, which has enhanced our work.

Reviewer #2 (Remarks to the Author):

Thank you to the authors for their revision. The vast majority of my comments have been adequately addressed although there remain some outstanding issues:

Methods:
- three-stage cluster-random survey should be instead three stage survey design with random selection of EAs and households.

This has been changed

- children as young as 1 yrs old, had a full blood draw? And was it a full blood draw or only serum using specialized tubes? Volume taken? More information is needed

Yes, venipuncture was undertaken in all participants. Phlebotomy and sample volume details have been added.

Discussion
- the first paragraph needs to summarize the key findings/take home messages and remove the comparison to other studies - this should go elsewhere in the discussion

Thank you for this comment, we have added key findings to the first paragraph and have separated it from the comparison to other studies
