# Supplementary material for: Sero-catalytic and spatial modeling of dengue virus force of infection in Timor-Leste
Source: Nat Commun. 2026 Sep 15;17:10815. doi: 10.1038/s41467-026-76716-9 (PMC13669370; doi:10.1038/s41467-026-76716-9)
Supplement: Supplementary file 3 [file 41467_2026_76716_MOESM1_ESM.docx]

**Sero-catalytic and spatial modeling of dengue virus force of infection in Timor-Leste**

Paul Arkell^1,2,*$^, Filipe de Neri Machado^3,4,*^, Anna Vicco^5,*^, Victoria M Cox^5,*^, Maria Y Tanesi^1^, Deolindo Ximenes^1^, Wes Hinsley^5^, Frederico Bosco^3,4^, Nelia Gomes^1^, Tessa Oakley^1^, Vanessa Solano^6^, Sarah L Sheridan^7^, Michael David^8,9^, Salvador Amaral^1^, Luzia Freitas^5,10^, Anthony DK Draper^1,11,12^, Nevio Sarmento^1^, Endang Soares da Silva^13^, Lucsendar Alves^1^, Neil Ferguson^5^, Christl A Donnelly^14, 15^, Jennifer Yan^1,+^, Joshua R Francis^1,+^, Ilaria Dorigatti^5,+^ , Nelson Martins^1 +^

1.	Global and Tropical Health Division, Menzies School of Health Research, Charles Darwin University, Dili, Timor-Leste

2.	Department of Infectious Diseases, Faculty of Medicine, Imperial College London, UK

3.	Instituto Nacional de Saude Publica de Timor-Leste, Timor-Leste Ministry of Health, Dili, Timor-Leste

4.	Departmanto Vigilancia no Epidemiojia, Timor-Leste Ministry of Health, Dili, Timor-Leste.

5.	MRC Centre for Global Infectious Disease Analysis, Department of Infectious Disease Epidemiology, School of Public Health, Imperial College London, UK

6.	Research Institute for the Environment and Livelihoods, Charles Darwin University, Darwin, Australia

7.	National Centre for Immunisation Research and Surveillance (NCIRS), Westmead, NSW, Australia

8.	Daffodil Centre, The University of Sydney, a joint venture with Cancer Council New South Wales, Sydney, NSW, Australia

9.	School of Medicine & Dentistry, Griffith University, Gold Coast, QLD, Australia

10.	Infectious Diseases Data Observatory, Oxford University, UK

11.	Northern Territory Centre for Disease Control, Public Health Division, NT Health, Darwin, Australia

12.	National Centre for Epidemiology and Population Health, Australian National University, Canberra, Australia

13.	Laboratório Nacional da Saúde, Dili, Timor-Leste

14.	Department of Statistics, University of Oxford, UK

15. Pandemic Sciences Institute, University of Oxford, UK

$	Corresponding author

*	These authors contributed equally

+	These authors jointly supervised this work

**Corresponding author**

Dr Paul Arkell

Email: paul.arkell@menzies.edu.au

**Supplementary Methods**

**Sensitivity analysis on the hierarchical catalytic model**

We performed a sensitivity analysis of the catalytic model described in the paper to assess the urban effect on the FOI, by including a urban effect $\gamma_{v}$(Model 2).

$\text{logit}(\lambda_{v})=\mu_{\text{v}} = \mu_{\text{n}}+\delta_{v}\text{ }\text{ + }\gamma_{v}X_{v}\text{ }\text{ }\text{ }\text{ }$	Equation S1

where $X_{v}$ is a binary indicator for village type (urban = 1, rural = 0) and $\gamma_{v}$ quantifies the fixed effect for urban villages (having taken rural as reference). We assumed $\gamma_{v}$yo be distributed as $\mathcal{N}(0,1).$

Model 1 and Model 2 performed similarly according to the statistical measurement LOOIC (leave-one-out cross-validation (LOO) information criterion) (2661.9 vs 2661.3). In Model 2, the FOI estimates were generally higher or equal to Model 1 (Figure S1). The estimated urban logit effect ($\gamma_{v}$) of Model 2 was 1.06 (95% CrI: 0.61, 1.56), suggesting no statistically significant difference in dengue FOI between urban and rural villages.

**Figure S1. Estimated FOI per EA (**$\boldsymbol{\lambda}_{\boldsymbol{v}}$**) by Model 1 and 2.** Estimated median (point) and 95% CrI (error bar) of the total FOI by EA, coloured by model type (Model 1 in orange and Model 2 in light blue).

**Sensitivity analysis at the municipality-level**

We performed a sensitivity analysis of the catalytic model described in the paper to assess the variation in the FOI at a lower spatial resolution (municipality-level). The FOI estimates ranged from 0.016 (95% CrI: 0.011, 0.023) in Aileu to 0.332 (95% CrI: 0.277, 0.400) in Dili.

**Table S1 Municipality-level FOI estimates.** The table reports the median and 95% CrI of the municipality-level FOI.

| **Municipality** | **median** | **ciL** | **ciU** |
| --- | --- | --- | --- |
| Aileu | 0.016 | 0.011 | 0.023 |
| Ainaro | 0.061 | 0.046 | 0.081 |
| Baucau | 0.220 | 0.170 | 0.285 |
| Bobonaro | 0.201 | 0.152 | 0.266 |
| Covalima | 0.090 | 0.069 | 0.116 |
| Dili | 0.332 | 0.277 | 0.400 |
| Ermera | 0.039 | 0.030 | 0.050 |
| Lautem | 0.218 | 0.161 | 0.297 |
| Liquica | 0.115 | 0.083 | 0.161 |
| Manatuto | 0.110 | 0.080 | 0.151 |
| Manufahi | 0.060 | 0.042 | 0.085 |
| Oecusse | 0.130 | 0.098 | 0.173 |
| Viqueque | 0.138 | 0.104 | 0.185 |

**Figure S2. Model fit of the observed seroprevalence data in each municipality in Timor-Leste.** The observed seroprevalence is reported in red (points) with the error bars representing the 95% exact binomial confidence interval. Individuals have been grouped into age-groups of 10 years. The estimated seroprevalence is reported in green, with the median shown as a line and the 95% CrI as a shaded area. The x axis represents the age in years, while the y axis represents the seroprevalence.

**Figure S3. Estimated median (point) and 95% CrI (error bar) of the total FOI by EA and municipality.** Each EA-level FOI is plotted as black points (median) with the error bars representing the 95% CrI, the municipality level FOI is plotted as red line (median) and red shaded area (05% CrI). Each box represents one municipality and the EA within.

**Sensitivity analysis on borderline IgG results**

We performed a sensitivity analysis of the catalytic model described in the paper to assess the impact of removing borderline IgG ELISA results (previously included and considered negative results). In total there were 57 borderline IgG ELISA results. The model was independently fitted with the two datasets (with and without the IgG borderline results). The FOI estimates from the two set of results were similar when the uncertainty around the FOI was smaller, while for those FOI with large uncertainty excluding IgG borderline results increases slightly the median FOI estimates.

**Figure S4.** **Estimated FOI per EA (**$\boldsymbol{\lambda}_{\boldsymbol{v}}$**) excluding borderline results.** Estimated median (point) and 95% CrI (error bar) of the total FOI by EA, coloured by model type (excluding borderline results in orange and including them in light blue).

**Meteorological data**

Monthly mean maximum temperature (degrees Celsius), mean minimum temperature (degrees Celsius), cumulative precipitation (mm), and mean vapor pressure (kPa) between January 2011 to December 2022 were extracted from TerraClimate as 5kmresolution [1], [2]. Relative humidity, $RH$, was calculated from the air temperature, $T$, and air vapour pressure, $e_{a}$, following Equation S1 which first calculates the saturation vapour pressure $e_{s}$[3]. The $RH$ was calculated at both minimum and maximum temperature. Table S1 describes the resolution and source of the meteorological, population, and environmental data.

$$e_{s}=0.611e^{\frac{17.27*T}{237.3+T}}$$

$$RH=100\frac{e_{a}}{e_{s}}$$

Equation S1

**Spatial random fields**

Spatial regression models were fitted to the posterior distributions of the 102 EA-level FOI estimated from the sero-catalytic model, using the stochastic partial differential equations (SPDE) approach in *R-INLA* [6]–[8]. The dengue FOI was assumed to be a spatially-correlated continuous process (a Gaussian field with a Matérn covariance function) which can be represented as a discrete process (Gaussian Markov Random Field, $\mu$, herein called field).

To support the assumption of spatial correlation we assigned each EA its nearest six neighbours, based on distance between the centroid coordinates, and computed the Moran I statistic for the DENV FOI at the coordinates using the *spdep* R package [9]; which indicates presence of spatial autocorrelation when the values are positive with significant p-values (Moran I statistic = 0.27. p-value = 1.585e-08).

The field is built on a triangulated mesh covering the coordinates of the EA centroids. The field is combined with an intercept ($\alpha$) to model the log-transformed median FOI ($\lambda$) per enumeration area $l$ as shown in equation S2:

${log(\lambda}_{l})=\alpha+\mu_{l}$

Equation S2

The field $\mu$ is the stationary solution to the SPDE which specifies the spatial correlation between two locations $i$ and $j$, and is characterised by the Matérn covariance function which specifies the distance $\delta$ at which $i$ and $j$ are spatially independent and includes a smoothing parameter $v$ and a scaling parameter $\kappa$. Following Lindgren *et al*., 2011, for all values of $v$,$\delta$ can be represented empirically by $\delta= \sqrt{\frac{8v}{\kappa}}$. [10]

We set penalised complexity prior distributions (pc-priors) on the Matérn function hyperparameters (using the *inla.spde2.pcmatern* function) which allow one to describe the prior distribution using probability statements (P) [11]. The prior distribution on $\delta$ was set as P(0.001, 0.1) which specifies that there is a probability smaller than 10% that the spatial range is less than 0.001 degrees (which is the approximate minimum distance between any two EA centroid coordinates). The prior distribution on $v$ was set as P(1, 0.10) which states that there is a probability smaller than 10% that the standard deviation of the spatial range is greater than one.

Several meshes were built and a spatial model was fitted to the median estimated FOI using each mesh. The models were compared visually and using the Watanabe-Akaike Information Criterion (WAIC) scores of the spatial models fitted to the data using each mesh (WAIC is a measure of predictive error where a lower score demonstrates a better model fit). For each mesh (Table S2, Figure S5), we specified the minimum angle of the triangles, the offset (which defines the extension of the inner and outer domain), and the maximum edge length of the triangles for the inner and outer domain. We specified meshes A-F using a default boundary around the centroid coordinates of the sampled EAs. We observed that a minimum angle of 25 degrees in mesh A gives medium-sized triangles compared to B and C, which are too small and large respectively, and that increasing the offset in E and F generates a better mesh than in A-D which have singular and irregular triangles in the outer domain (Figure S5). In meshes A-F the boundary extends far into the ocean, so for mesh G and H the boundary was specified using the coordinates of the country outline, to separate the field for Atauro island and Oecusse which are non-continuous with the main island. For mesh H, the cut-off distance was set to 0.02 to build smaller triangles at the boundary edge than for mesh H. The WAIC scores of mesh G and H were 269.72 and 269.44 respectively, so mesh H was used as the preferred mesh in the rest of the analysis.

**Supplementary Tables**

**Table S2: Prior distribution of estimated parameters used in catalytic models.**

| **Parameter** | **Description** | **Prior** |
| --- | --- | --- |
| $\delta_{v}$ | village-level deviation from the national FOI | $\mathcal{N}(0,\sigma_{v})$ |
| $\delta_{h}$ | Household-level deviation from the village FOI | $\mathcal{N}(0,\sigma_{h})$ |
| $\sigma_{v}$ | Single SD of the village-level deviation from the national FOI | Half-Normal(0.1, 0.1) |
| $\sigma_{h}$ | Single SD of the household-level deviation from the village FOI | Half-Normal(0.1, 0.1) |
| $se$ | Test sensitivity ( $0<se<1$) | $\mathcal{N}(0.77,0.1)$ |
| $sp$ | Test specificity ( $0<sp<1$) | $\mathcal{N}(0.93,0.1)$ |

**Table S3. Enumeration area (EA)-, household- and individual- participation rates in each municipality**

|  | **Participating EAs** | **Non-participating EAs** | **EA participation rate** | **Participating households** | **Non-participating households** | **Household participation rate** | **Participants** | **Non-participating household occupants** | **Individual participation rate** |
| --- | --- | --- | --- | --- | --- | --- | --- | --- | --- |
| **Aileu** | 4 | 2 | **66.7%** | 67 | 48 | 58.3% | 184 | 153 | 54.6% |
| **Ainaro** | 6 | 0 | **100.0%** | 101 | 39 | 72.1% | 286 | 145 | 66.4% |
| **Baucau** | 11 | 0 | **100.0%** | 220 | 34 | 86.6% | 451 | 356 | 55.9% |
| **Bobonaro** | 8 | 0 | **100.0%** | 139 | 46 | 75.1% | 388 | 121 | 76.2% |
| **Covalima** | 6 | 0 | **100.0%** | 124 | 14 | 89.9% | 356 | 205 | 63.5% |
| **Dili** | 30 | 0 | **100.0%** | 437 | 233 | 65.2% | 1205 | 1471 | 45.0% |
| **Ermera** | 7 | 5 | **58.3%** | 115 | 165 | 41.1% | 329 | 235 | 58.3% |
| **Lautem** | 5 | 1 | **83.3%** | 104 | 37 | 73.8% | 325 | 184 | 63.9% |
| **Liquica** | 5 | 1 | **83.3%** | 80 | 82 | 49.4% | 226 | 190 | 54.3% |
| **Manatuto** | 4 | 0 | **100.0%** | 85 | 11 | 88.5% | 246 | 75 | 76.6% |
| **Manufahi** | 4 | 1 | **80.0%** | 56 | 58 | 49.1% | 178 | 103 | 63.3% |
| **Oecusse** | 6 | 0 | **100.0%** | 128 | 26 | 83.1% | 302 | 220 | 57.9% |
| **Viqueque** | 6 | 1 | **85.7%** | 99 | 65 | 60.4% | 304 | 189 | 61.7% |
| **Grand Total** | **102** | **11** | **90.3%** | **1755** | 858 | 67.2% | **4780** | **3647** | 56.7% |

**Table S4: Demographic details of participants in each municipality**

|  | **Male** | **% Male** | **Female** | **% Female** | **Median age** | **Minimum age** | **Maximum age** | **Age quartile 1** | **Age quartile 3** | **Child** | **% Child** | **Adult** | **% Adult** | **Rural household** | **% Rural household** | **Urban household** | **% Urban household** | **Dengue IgG negative** | **% Dengue IgG negative** | **Dengue IgG positive** | **% Dengue IgG positive** |
| --- | --- | --- | --- | --- | --- | --- | --- | --- | --- | --- | --- | --- | --- | --- | --- | --- | --- | --- | --- | --- | --- |
| **Aileu** | 73 | 39.7% | 111 | 60.3% | 28 | 5 | 77 | 17 | 52 | 38 | 20.7% | 146 | 79.3% | 184 | 100.0% | 0 | 0.0% | 109 | 59.2% | 75 | 40.8% |
| **Ainaro** | 122 | 42.7% | 164 | 57.3% | 23 | 1 | 85 | 11 | 43 | 104 | 36.4% | 182 | 63.6% | 239 | 83.6% | 47 | 16.4% | 100 | 35.0% | 186 | 65.0% |
| **Baucau** | 172 | 38.1% | 279 | 61.9% | 32 | 1 | 85 | 16 | 51 | 111 | 24.6% | 340 | 75.4% | 360 | 79.8% | 91 | 20.2% | 43 | 9.5% | 408 | 90.5% |
| **Bobonaro** | 161 | 41.5% | 227 | 58.5% | 31 | 1 | 87 | 15 | 46 | 98 | 25.3% | 290 | 74.7% | 388 | 100.0% | 0 | 0.0% | 34 | 8.8% | 354 | 91.2% |
| **Covalima** | 137 | 38.5% | 219 | 61.5% | 30 | 1 | 89 | 12 | 49 | 109 | 30.6% | 247 | 69.4% | 356 | 100.0% | 0 | 0.0% | 81 | 22.8% | 275 | 77.2% |
| **Dili** | 482 | 40.0% | 723 | 60.0% | 23 | 1 | 102 | 12 | 38 | 367 | 30.5% | 838 | 69.5% | 307 | 25.5% | 898 | 74.5% | 72 | 6.0% | 1133 | 94.0% |
| **Ermera** | 147 | 44.7% | 182 | 55.3% | 30 | 1 | 88 | 15 | 46.25 | 87 | 26.4% | 242 | 73.6% | 295 | 89.7% | 34 | 10.3% | 136 | 41.3% | 193 | 58.7% |
| **Lautem** | 135 | 41.5% | 190 | 58.5% | 34 | 1 | 93 | 11 | 53 | 121 | 37.2% | 204 | 62.8% | 325 | 100.0% | 0 | 0.0% | 35 | 10.8% | 290 | 89.2% |
| **Liquica** | 90 | 39.8% | 136 | 60.2% | 30 | 1 | 90 | 15 | 51 | 57 | 25.2% | 169 | 74.8% | 183 | 81.0% | 43 | 19.0% | 41 | 18.1% | 185 | 81.9% |
| **Manatuto** | 95 | 38.6% | 151 | 61.4% | 27 | 1 | 85 | 11 | 46 | 88 | 35.8% | 158 | 64.2% | 169 | 68.7% | 77 | 31.3% | 57 | 23.2% | 189 | 76.8% |
| **Manufahi** | 69 | 38.8% | 109 | 61.2% | 26 | 1 | 81 | 10 | 45.25 | 65 | 36.5% | 113 | 63.5% | 178 | 100.0% | 0 | 0.0% | 64 | 36.0% | 114 | 64.0% |
| **Oecusse** | 137 | 45.4% | 165 | 54.6% | 32 | 1 | 94 | 14 | 49 | 80 | 26.5% | 222 | 73.5% | 240 | 79.5% | 62 | 20.5% | 47 | 15.6% | 255 | 84.4% |
| **Viqueque** | 126 | 41.4% | 178 | 58.6% | 29 | 2 | 90 | 14.25 | 47.75 | 84 | 27.6% | 220 | 72.4% | 275 | 90.5% | 29 | 9.5% | 44 | 14.5% | 260 | 85.5% |
| **Grand Total** | **1946** | **40.7%** | **2834** | **59.3%** | **28** | **1** | **102** | **13** | **46** | **1409** | **29.5%** | **3371** | **70.5%** | **3499** | **73.2%** | **1281** | **26.8%** | **863** | **18.1%** | **3917** | **81.9%** |

**Table S5: Force of infection (FOI) estimates by enumeration area (EA) from the sero-catalytic model. Longitude and latitude of each EA centroid is also shown.**

| **EA name** | **FOI (95% CrI)** | **Mun-icipality** | **Sub-municipality** | **Suco** | **Longitude** | **Latitude** |
| --- | --- | --- | --- | --- | --- | --- |
| 10103022 | 0.020 (0.013, 0.030) | Aileu | Aileu Vila | Fahiria | 125.5911 | -8.72665 |
| 10104012 | 0.026 (0.016, 0.041) | Aileu | Aileu Vila | Fatubossa | 125.5638 | -8.81222 |
| 10104032 | 0.010 (0.005, 0.018) | Aileu | Aileu Vila | Fatubossa | 125.5421 | -8.82593 |
| 10111042 | 0.016 (0.009, 0.025) | Aileu | Aileu Vila | Suco Liurai | 125.5216 | -8.76602 |
| 20101051 | 0.047 (0.030, 0.073) | Ainaro | Ainaro | Ainaro | 125.4992 | -8.98757 |
| 20103012 | 0.080 (0.054, 0.119) | Ainaro | Ainaro | Manutaci | 125.5197 | -8.98526 |
| 20201072 | 0.045 (0.029, 0.074) | Ainaro | Hato-Udo | Foho-Ai-Lico | 125.6615 | -9.14524 |
| 20202092 | 0.212 (0.124, 0.372) | Ainaro | Hato-Udo | Leolima | 125.5784 | -9.13306 |
| 20202112 | 0.027 (0.015, 0.045) | Ainaro | Hato-Udo | Leolima | 125.5422 | -9.19143 |
| 20408062 | 0.017 (0.009, 0.031) | Ainaro | Maubisse | Maubisse | 125.5823 | -8.83708 |
| 30107042 | 0.064 (0.039, 0.106) | Baucau | Baguia | Lavateri | 126.6726 | -8.56643 |
| 30201031 | 0.261 (0.125, 0.565) | Baucau | Baucau | Bahu | 126.4522 | -8.4561 |
| 30203082 | 0.292 (0.107, 0.729) | Baucau | Baucau | Buibau | 126.4641 | -8.51051 |
| 30208042 | 0.459 (0.210, 0.805) | Baucau | Baucau | SeiÃ§al | 126.5248 | -8.51821 |
| 30209121 | 0.237 (0.133, 0.447) | Baucau | Baucau | Tirilolo | 126.4623 | -8.46649 |
| 30302012 | 0.212 (0.103, 0.480) | Baucau | Laga | Libagua | 126.6561 | -8.4927 |
| 30303012 | 0.429 (0.218, 0.748) | Baucau | Laga | Nunira | 126.6294 | -8.46225 |
| 30305042 | 0.086 (0.052, 0.146) | Baucau | Laga | Sagadate | 126.6853 | -8.53214 |
| 30305082 | 0.167 (0.081, 0.366) | Baucau | Laga | Sagadate | 126.7171 | -8.51676 |
| 30406022 | 0.423 (0.191, 0.790) | Baucau | Quelicai | GuruÃ§Ã | 126.6083 | -8.57476 |
| 30602022 | 0.080 (0.048, 0.133) | Baucau | Venilale | Baha Mori | 126.4297 | -8.60586 |
| 40101092 | 0.145 (0.084, 0.266) | Bobonaro | Atabae | Aidabaleten | 125.0995 | -8.85126 |
| 40103022 | 0.420 (0.188, 0.789) | Bobonaro | Atabae | Hataz | 125.164 | -8.87183 |
| 40104062 | 0.117 (0.053, 0.559) | Bobonaro | Atabae | Rairobo | 125.1716 | -8.80121 |
| 40203052 | 0.121 (0.070, 0.223) | Bobonaro | Balibo | Cowa | 125.0106 | -8.99911 |
| 40205042 | 0.071 (0.044, 0.114) | Bobonaro | Balibo | Leolima | 125.0644 | -8.90637 |
| 40303012 | 0.108 (0.048, 0.257) | Bobonaro | Bobonaro | Bobonaro | 125.3485 | -9.04467 |
| 40403012 | 0.519 (0.295, 0.816) | Bobonaro | Cailaco | Goulolo | 125.2778 | -8.92728 |
| 40604032 | 0.151 (0.090, 0.265) | Bobonaro | Maliana | Raifun | 125.208 | -8.97344 |
| 50101012 | 0.021 (0.014, 0.031) | Covalima | Fatululic | Fatululic | 125.1161 | -9.20652 |
| 50203022 | 0.031 (0.02, 0.047) | Covalima | Fatumean | Nanu | 125.0136 | -9.28893 |
| 50501092 | 0.301 (0.175, 0.517) | Covalima | Suai | Beco | 125.3901 | -9.19959 |
| 50703042 | 0.368 (0.207, 0.640) | Covalima | Zumalai | Lour | 125.3892 | -9.07868 |
| 50705062 | 0.107 (0.070, 0.169) | Covalima | Zumalai | Raimea | 125.512 | -9.16869 |
| 50708032 | 0.126 (0.079, 0.201) | Covalima | Zumalai | Zulo | 125.46 | -9.15913 |
| 60102042 | 0.201 (0.101, 0.422) | Dili | Atauro | Biqueli | 125.6022 | -8.16681 |
| 60103042 | 0.401 (0.165, 0.796) | Dili | Atauro | Macadade | 125.5427 | -8.28541 |
| 60201012 | 0.244 (0.164, 0.377) | Dili | Cristo Rei | Balibar | 125.5865 | -8.59254 |
| 60202031 | 0.315 (0.159, 0.617) | Dili | Cristo Rei | Becora | 125.6024 | -8.55976 |
| 60202131 | 0.378 (0.196, 0.704) | Dili | Cristo Rei | Becora | 125.5984 | -8.56315 |
| 60202201 | 0.147 (0.079, 0.478) | Dili | Cristo Rei | Becora | 125.6128 | -8.56887 |
| 60203071 | 0.241 (0.124, 0.466) | Dili | Cristo Rei | Bidau Santana | 125.5962 | -8.55217 |
| 60204051 | 0.471 (0.226, 0.817) | Dili | Cristo Rei | Camea | 125.6253 | -8.56696 |
| 60204101 | 0.372 (0.136, 0.780) | Dili | Cristo Rei | Camea | 125.614 | -8.56376 |
| 60204212 | 0.145 (0.081, 0.274) | Dili | Cristo Rei | Camea | 125.6383 | -8.57996 |
| 60206081 | 0.446 (0.266, 0.721) | Dili | Cristo Rei | Hera | 125.6566 | -8.53594 |
| 60301091 | 0.180 (0.082, 0.633) | Dili | Dom Aleixo | Bairro Pite | 125.5602 | -8.55958 |
| 60301161 | 0.210 (0.120, 0.379) | Dili | Dom Aleixo | Bairro Pite | 125.5541 | -8.56136 |
| 60301241 | 0.117 (0.058, 0.251) | Dili | Dom Aleixo | Bairro Pite | 125.5624 | -8.56192 |
| 60301261 | 0.142 (0.070, 0.344) | Dili | Dom Aleixo | Bairro Pite | 125.5633 | -8.56225 |
| 60301271 | 0.225 (0.115, 0.534) | Dili | Dom Aleixo | Bairro Pite | 125.5647 | -8.56278 |
| 60301301 | 0.257 (0.130, 0.510) | Dili | Dom Aleixo | Bairro Pite | 125.5618 | -8.56286 |
| 60301351 | 0.493 (0.239, 0.835) | Dili | Dom Aleixo | Bairro Pite | 125.5559 | -8.57114 |
| 60301421 | 0.304 (0.116, 0.735) | Dili | Dom Aleixo | Bairro Pite | 125.5358 | -8.58389 |
| 60302141 | 0.216 (0.106, 0.610) | Dili | Dom Aleixo | Comoro | 125.5174 | -8.54946 |
| 60302201 | 0.264 (0.119, 0.588) | Dili | Dom Aleixo | Comoro | 125.5114 | -8.56717 |
| 60302251 | 0.370 (0.160, 0.762) | Dili | Dom Aleixo | Comoro | 125.5335 | -8.54968 |
| 60302301 | 0.195 (0.123, 0.316) | Dili | Dom Aleixo | Comoro | 125.534 | -8.55257 |
| 60302521 | 0.240 (0.130, 0.579) | Dili | Dom Aleixo | Comoro | 125.5309 | -8.55841 |
| 60302641 | 0.100 (0.035, 0.323) | Dili | Dom Aleixo | Comoro | 125.5356 | -8.56578 |
| 60402012 | 0.185 (0.100, 0.356) | Dili | Metinaro | Sabuli | 125.7285 | -8.53157 |
| 60506031 | 0.226 (0.112, 0.455) | Dili | Nain Feto | Santa Cruz | 125.5816 | -8.55988 |
| 60603022 | 0.449 (0.220, 0.810) | Dili | Vera Cruz | Dare | 125.5549 | -8.58741 |
| 60604041 | 0.217 (0.124, 0.398) | Dili | Vera Cruz | Lahane Ocidental | 125.5783 | -8.57894 |
| 60605021 | 0.112 (0.065, 0.196) | Dili | Vera Cruz | Mascarenhas | 125.5799 | -8.56463 |
| 70109012 | 0.049 (0.030, 0.080) | Ermera | Atsabe | Malabe | 125.3874 | -8.92122 |
| 70205032 | 0.019 (0.012, 0.030) | Ermera | Ermera | Mertuto | 125.3971 | -8.76521 |
| 70207022 | 0.039 (0.027, 0.055) | Ermera | Ermera | Ponilala | 125.3753 | -8.71904 |
| 70210031 | 0.089 (0.052, 0.148) | Ermera | Ermera | Talimoro | 125.4339 | -8.72779 |
| 70305052 | 0.018 (0.010, 0.030) | Ermera | Hatulia | Fatubolo | 125.372 | -8.75788 |
| 70506042 | 0.042 (0.026, 0.065) | Ermera | Railaco | Railaco Leten | 125.507 | -8.69794 |
| 70509022 | 0.049 (0.030, 0.080) | Ermera | Railaco | Tocoluli | 125.4202 | -8.71017 |
| 80203032 | 0.402 (0.228, 0.682) | Lautem | LautÃm | Daudere | 126.826 | -8.46209 |
| 80301062 | 0.205 (0.084, 0.562) | Lautem | Lospalos | Bauro | 127.0091 | -8.42621 |
| 80307032 | 0.206 (0.134, 0.307) | Lautem | Lospalos | Lore 2 | 126.9917 | -8.55239 |
| 80405022 | 0.068 (0.044, 0.105) | Lautem | Luro | Luro | 126.8418 | -8.55867 |
| 80502042 | 0.234 (0.131, 0.417) | Lautem | Tutuala | Tutuala | 127.2432 | -8.44354 |
| 90104032 | 0.045 (0.027, 0.074) | Liquica | Bazartete | Leorema | 125.3831 | -8.6597 |
| 90106012 | 0.059 (0.041, 0.085) | Liquica | Bazartete | Metagou | 125.3671 | -8.64346 |
| 90107012 | 0.359 (0.160, 0.725) | Liquica | Bazartete | Motaulun | 125.4131 | -8.57641 |
| 90109042 | 0.132 (0.059, 0.509) | Liquica | Bazartete | Ulmera | 125.4556 | -8.58413 |
| 90203061 | 0.320 (0.148, 0.762) | Liquica | LiquiÃÃ¡ | Dato | 125.3358 | -8.59753 |
| 100204012 | 0.252 (0.134, 0.499) | Manatuto | Laclo | Uma Naruc | 125.9054 | -8.55879 |
| 100301042 | 0.049 (0.032, 0.073) | Manatuto | Laclubar | Batara | 125.9519 | -8.70867 |
| 100502011 | 0.191 (0.120, 0.311) | Manatuto | Manatuto | Aiteas | 126.0385 | -8.5159 |
| 100602022 | 0.054 (0.035, 0.084) | Manatuto | Soibada | Leo-Hat | 125.967 | -8.8575 |
| 110102032 | 0.023 (0.009, 0.047) | Manufahi | Alas | Dotik | 125.8308 | -8.96764 |
| 110105012 | 0.133 (0.086, 0.207) | Manufahi | Alas | Uma Berloic | 125.9276 | -9.12137 |
| 110201032 | 0.032 (0.021, 0.048) | Manufahi | Fatuberlio | Bubussuso | 125.7666 | -8.89974 |
| 110307012 | 0.042 (0.024, 0.072) | Manufahi | Same | Rotuto | 125.6011 | -8.97127 |
| 120201032 | 0.146 (0.082, 0.323) | Oecusse | Oesilo | Bobometo | 124.3554 | -9.30304 |
| 120201052 | 0.139 (0.086, 0.232) | Oecusse | Oesilo | Bobometo | 124.3792 | -9.32518 |
| 120302081 | 0.404 (0.203, 0.720) | Oecusse | Pante Macassar | Costa | 124.3427 | -9.20812 |
| 120308082 | 0.122 (0.072, 0.226) | Oecusse | Pante Macassar | Taiboco | 124.2832 | -9.29483 |
| 120401032 | 0.027 (0.014, 0.048) | Oecusse | Passabe | Abani | 124.3378 | -9.43847 |
| 120401132 | 0.062 (0.039, 0.100) | Oecusse | Passabe | Abani | 124.3278 | -9.46321 |
| 130501032 | 0.103 (0.061, 0.191) | Viqueque | Viqueque | Bahalarauain | 126.2705 | -8.91203 |
| 130502062 | 0.070 (0.048, 0.102) | Viqueque | Viqueque | Bibileo | 126.3101 | -8.93673 |
| 130503031 | 0.123 (0.067, 0.234) | Viqueque | Viqueque | Caraubalo | 126.3673 | -8.86818 |
| 130505072 | 0.151 (0.090, 0.260) | Viqueque | Viqueque | Luca | 126.2574 | -8.92059 |
| 130509042 | 0.119 (0.068, 0.205) | Viqueque | Viqueque | Uma Uain Craic | 126.3781 | -8.87941 |
| 130509062 | 0.213 (0.130, 0.358) | Viqueque | Viqueque | Uma Uain Craic | 126.3879 | -8.89039 |

**Table S6: Meteorological, environmental, and demographic variables.**

| Variable | Source | Spatial resolution | Temporal resolution | Number of datapoints |
| --- | --- | --- | --- | --- |
| Middle infrared reflectance (MIR) | MODIS | 1km | 8 days | 14057311 |
| Enhanced vegetation index (EVI) | MODIS | 1km | 8 days | 14057311 |
| Precipitation | TerraClimate | 5km | 1 month | 166752 |
| Maximum air temperature at ground level during 24-hour period (Tmax) | TerraClimate | 5km | 1 month | 166752 |
| Minimum air temperature at ground level during 24-hour period (Tmin) | TerraClimate | 5km | 1 month | 166752 |
| Relative humidity at Tmax | TerraClimate | 5km | 1 month | 166752 |
| Relative humidity at Tmin | TerraClimate | 5km | 1 month | 166752 |
| Elevation | Worldclim | 1km | NA | 25157 |
| Population | Landscan | 1 km | 1 year | 234546 |

**Table S7. Parameters used to build triangulated meshes for the spatial field.**

| Mesh | Minimum angle (degrees) | Maximum edge length | Offset | Boundary | Coordinates | Cut-off distance | WAIC |
| --- | --- | --- | --- | --- | --- | --- | --- |
| A | 25 | 0.2, 0.4 | 0.2, 0.3 | null | EA centroids | 0.03 | 241.52 |
| B | 35 | 0.2, 0.4 | 0.2, 0.3 | null | EA centroids | 0.03 | 218.27 |
| C | 15 | 0.2, 0.4 | 0.2, 0.3 | null | EA centroids | 0.03 | 242.56 |
| D | 25 | 0.5, 0.5 | 0.2, 0.3 | null | EA centroids | 0.03 | 245.36 |
| E | 25 | 0.2, 0.4 | 0.2, 0.6 | null | EA centroids | 0.03 | 241.52 |
| F | 25 | 0.2, 0.4 | 0.6, 0.6 | null | EA centroids | 0.03 | 241.16 |
| G | 25 | 0.2, 0.4 | 0.2, 0.6 | country boundary | null | 0.03 | 269.72 |
| H | 25 | 0.2, 0.4 | 0.2, 0.6 | country boundary | null | 0.02 | 269.44 |

**Supplementary Figures**

**Figure S5. Histogram showing raw IgG antibody titres from the serosurvey.** A dashed vertical line is shown to represent the cutoff used to classify the samples as seronegative or seropositive.

**Figure S6: Enumeration areas of Timor-Leste, in black, overlaid with points showing the centroids of the 5km-resolution meteorological data, in red.**

**Figure S7: Maps of the average (A) maximum temperature, (B) minimum temperature, (C) relative humidity at minimum temperature, (D) relative humidity at maximum temperature, and (E) precipitation for each enumeration area in Timor-Leste.**

**Figure S8: Maps of the average (A) Enhanced Vegetation Index (EVI), (B) Mid infra-red reflectance (MIR), (C) elevation above sea level, and (D) population on the log scale for each enumeration area in Timor-Leste.**

**Figure S9: Triangulated meshes tested in the analysis, with Timor-Leste shown in light blue, the coordinates of the centroids of the sampled enumeration areas represented as black points, and boundaries in dark blue.**

**Figure S10. Model fit of the observed seroprevalence data in each EA in Timor-Leste.** The observed seroprevalence is reported in red (points) with the error bars representing the 95% exact binomial confidence interval. Individuals have been grouped into age-groups of 10 years. The estimated seroprevalence is reported in green, with the median shown as a line and the 95% CrI as a shaded area. The x axis represents the age in years, while the y axis represents the seroprevalence. Each panel represents a different EA identified by the number in the title.

**Figure S11: Collinearity of the meteorological and demographic variables.** The points are scaled and coloured with their Pearson’s correlation coefficient.

**Figure S12: Cross-validation of the spatial model. Median and 95% credible intervals of the ensemble spatial model dengue FOI estimates on the y-axis, compared to the median and 95% CrI of the sero-catalytic model dengue FOI estimates on the x-axis, for the 102 sampled enumeration areas.**

**Figure S13: Exceedance probability maps for the predicted seroprevalence at 9 years of age (serop9) across Timor-Leste.** The probability that the serop9 exceeds 60% (A) and 40% (B) are shown, and are extracted from the joint spatial model estimated posterior distributions.

**Supplementary References**

[1]	“TerraClimate.” [Online]. Available: https://www.climatologylab.org/terraclimate.html.

[2]	J. T. Abatzoglou, S. Z. Dobrowski, S. A. Parks, and K. C. Hegewisch, “TerraClimate, a high-resolution global dataset of monthly climate and climatic water balance from 1958–2015,” *Sci. Data 2018 51*, vol. 5, no. 1, pp. 1–12, Jan. 2018.

[3]	Alduchov A Oleg and Eskridge E Robert, “Improved Magnus Form Approximation of Saturation Vapor Pressure,” *J. Appl. Meteorol. Climatol.*, 1996. vol. 35, no. 4, pp.601-609

[4]	Statistics Department Timor-Leste Ministry of Infrastructure, “EA kml shapefile.” 2023.

[5]	D. Baston, “exactextractr: Fast Extraction from Raster Datasets using Polygons.” 2020.

[6]	H. Bakka *et al.*, “Spatial modeling with R-INLA: A review,” *Wiley Interdiscip. Rev. Comput. Stat.*, vol. 10, no. 6, pp. 1–33, 2018.

[7]	F. Lindgren, H. Rue, and J. Lindström, “An explicit link between Gaussian fields and Gaussian Markov random fields: the stochastic partial differential equation approach,” *J. R. Stat. Soc. Ser. B (Statistical Methodol.*, vol. 73, no. 4, pp. 423–498, Sep. 2011.

[8]	M. Blangiardo and M. Cameletti, *Spatial and Spatio-temporal Bayesian Models with R-INLA*. John Wiley & Sons Ltd, Chichester, United Kingdom. 2015.

[9]	R. Bivand *et al*, "spdep: Spatial Dependence: Weighting Schemes, Statistics". 2023

[10]	F. Lindgren and H. Rue, “An explicit link between Gaussian fields and Gaussian Markov random fields: the stochastic partial differential equation approach,” *J. R. Stat. Soc. Ser. B*, vol. 73, no. 4, pp. 423–498, 2011.

[11]	G. A. Fuglstad, D. Simpson, F. Lindgren, and H. Rue, “Constructing Priors that Penalize the Complexity of Gaussian Random Fields,” *https://doi.org/10.1080/01621459.2017.1415907*, vol. 114, no. 525, pp. 445–452, Jan. 2018.
